# Age-Related Neuronal Degeneration: Complementary Roles of Nucleotide Excision Repair and Transcription-Coupled Repair in Preventing Neuropathology

**DOI:** 10.1371/journal.pgen.1002405

**Published:** 2011-12-08

**Authors:** Dick Jaarsma, Ingrid van der Pluijm, Monique C. de Waard, Elize D. Haasdijk, Renata Brandt, Marcel Vermeij, Yvonne Rijksen, Alex Maas, Harry van Steeg, Jan H. J. Hoeijmakers, Gijsbertus T. J. van der Horst

**Affiliations:** 1 Department of Neuroscience, Erasmus University Medical Center, Rotterdam, The Netherlands; 2 Department of Genetics, Cancer Genomics Center, Erasmus University Medical Center, Rotterdam, The Netherlands; 3 Department of Pathology, Erasmus University Medical Center, Rotterdam, The Netherlands; 4 National Institute of Public Health and the Environment (RIVM), Laboratory of Toxicology, Pathology, and Genetics (TOX), Bilthoven, The Netherlands; Stanford University School of Medicine, United States of America

## Abstract

Neuronal degeneration is a hallmark of many DNA repair syndromes. Yet, how DNA damage causes neuronal degeneration and whether defects in different repair systems affect the brain differently is largely unknown. Here, we performed a systematic detailed analysis of neurodegenerative changes in mouse models deficient in nucleotide excision repair (NER) and transcription-coupled repair (TCR), two partially overlapping DNA repair systems that remove helix-distorting and transcription-blocking lesions, respectively, and that are associated with the UV-sensitive syndromes xeroderma pigmentosum (XP) and Cockayne syndrome (CS). TCR–deficient *Csa^−/−^* and *Csb^−/−^* CS mice showed activated microglia cells surrounding oligodendrocytes in regions with myelinated axons throughout the nervous system. This white matter microglia activation was not observed in NER–deficient *Xpa^−/−^* and *Xpc^−/−^* XP mice, but also occurred in *Xpd^XPCS^* mice carrying a point mutation (G602D) in the *Xpd* gene that is associated with a combined XPCS disorder and causes a partial NER and TCR defect. The white matter abnormalities in TCR–deficient mice are compatible with focal dysmyelination in CS patients. Both TCR–deficient and NER–deficient mice showed no evidence for neuronal degeneration apart from p53 activation in sporadic (*Csa^−/−^*, *Csb^−/−^*) or highly sporadic (*Xpa^−/−^*, *Xpc^−/−^*) neurons and astrocytes. To examine to what extent overlap occurs between both repair systems, we generated TCR–deficient mice with selective inactivation of NER in postnatal neurons. These mice develop dramatic age-related cumulative neuronal loss indicating DNA damage substrate overlap and synergism between TCR and NER pathways in neurons, and they uncover the occurrence of spontaneous DNA injury that may trigger neuronal degeneration. We propose that, while *Csa^−/−^* and *Csb^−/−^* TCR–deficient mice represent powerful animal models to study the mechanisms underlying myelin abnormalities in CS, neuron-specific inactivation of NER in TCR–deficient mice represents a valuable model for the role of NER in neuronal maintenance and survival.

## Author Summary

Metabolism produces reactive oxygen species that damage our DNA and other cellular components, and as such it contributes to the aging process, including neuronal degeneration. Accordingly, genetic disorders associated with impaired DNA damage repair are frequently associated with premature onset of aging pathology in a variety of tissues, including the brain. This is well-illustrated by the progeroid DNA repair syndromes xeroderma pigmentosum (XP) and Cockayne syndrome (CS), in which patients suffer from defects in nucleotide excision repair (NER) and transcription-coupled repair (TCR), two partially overlapping DNA repair systems that remove helix-distorting and transcription-blocking lesions, respectively. We have used a panel of XP and CS mice (including conditional double-mutant animals) to systematically investigate the impact of NER and TCR defects on neuronal degeneration. We have shown that, whereas a TCR defect causes white matter pathology, a NER defect can result in age related cumulative loss of neurons. These findings well match the neuropathology observed in CS and XP patients, underscoring the impact of spontaneous DNA damage in the onset of neuronal aging. Therefore, the XP and CS mouse models serve as valuable tools to delineate intervention strategies that combat age-associated pathology of the brain.

## Introduction

DNA is continuously damaged by spontaneous hydrolytic decay, endogenous metabolites (e.g. reactive oxygen species, malondialdehyde), and environmental genotoxins. DNA lesions can give rise to irreversible mutations and chromosomal aberrations that may trigger carcinogenesis. Alternatively, DNA damage can cause replicative senescence and cell death, which promotes the process of aging [1]. Cumulative DNA damage has also been implicated in the functional deterioration and degeneration of long-living post-mitotic cells such as neurons [2]. To counteract the harmful effects of DNA injuries, cells have a variety of DNA surveillance and repair systems. The importance of these genome maintenance pathways for human health is well illustrated by a heterogeneous set of inherited syndromes that are associated with defects in specific DNA repair pathways resulting in cancer predisposition, developmental abnormalities, accelerated aging and neurodevelopmental or neurodegenerative abnormalities [1], [3]–[5].

Nucleotide excision repair (NER) is a key DNA repair pathway for removal of UV-induced DNA damage and a wide range of other helix-distorting lesions, including bulky chemical adducts and specific types of oxidative damage [1]. In NER the DNA lesion is removed as a part of a 25–30 nucleotide single-strand fragment excised via a multi-step reaction followed by resynthesis of the excised strand [4], [6]–[8]. NER can be divided into two subpathways that differ in the damage recognition step: While global genome NER (GG-NER) removes distorting DNA damage throughout the genome, transcription-coupled NER (TC-NER) specifically targets transcription-blocking lesions in the template strand of active genes to allow recovery of transcription after damage induction [1], [4], [7], [8]. Several NER proteins have functions beyond NER, which is particularly evident for the transcription/repair factor TFIIH, which is required for the local opening of the damaged DNA in NER, but in addition plays an essential role in transcription. Furthermore, several lines of evidence indicate that TC-NER components are involved in repair of transcription-blocking lesions independent of the NER core complex, putatively via recruitment of other repair mechanisms. The term transcription-coupled repair (TCR) has been used to designate this broader, still poorly defined repair process [7], [9], [10].

NER gene defects are associated with a heterogeneous set of rare clinical syndromes, whose characteristics can be explained by the type of NER pathway that is affected or by defects in additional functions of these NER components in other DNA repair pathways or transcription. Selective defects in GG-NER, resulting from mutations in the *XPC* and *XPE* (also termed *UV-DDB2*) genes encoding GG-NER-specific damage recognition proteins, cause xeroderma pigmentosum (XP), a photosensitivity syndrome characterized by UV-hypersensitivity, pigmentation abnormalities and UV-induced skin cancer predisposition [11], [12]. Cancer-predisposition in XP-C patients is explained by bulky lesions that accumulate over the entire genome causing mutations after replication [1]. Selective defects in TC-NER result from mutations in the genes encoding the proteins CSA or CSB, both of which are selectively recruited to stalled RNA polymerase II [13]. Mutations in *CSB* and *CSA* are associated with Cockayne syndrome (CS), a progeroid disorder characterized by cachectic dwarfism and progressive neurological abnormalities, in addition to skin photosensitivity [14]–[16]. CS patients do not show cancer predisposition, which is explained by the normal function of GG-NER, and indicates that TC-NER is not required for preventing cancer. On the other hand, most CS pathological features cannot be explained by the sole loss of TC-NER function as they do not occur in XP-A patients, which show a combined GG-NER/TC-NER deficiency, resulting from mutations in the gene encoding for the core NER protein XPA. Thus XP-A patients present with UV-hypersensitivity and skin cancer predisposition, like XP-C patients, usually in combination with progressive neurological abnormalities (see below) [17]–[19], but they do not develop cachectic dwarfism and other progeroid features of CS patients. This has led to the notion that the CS phenotype is largely the consequence of an overall TCR defect, i.e., the inability to rescue transcription arrested by NER- and non-NER-types of DNA damage [7], [20], [21]. In addition it has been suggested that CS is associated with transcriptional abnormalities independent of DNA lesions [7], [22], [23].

The complementarity of NER and TCR DNA repair pathways and disorders resulting from deficiencies in these processes is also illustrated by XPCS patients, which display both XP and CS symptoms. XPCS is caused by mutations in the *XPB* or *XPD* genes, both encoding helicases of the transcription/repair factor TFIIH, or in the *XPG* gene, encoding the endonuclease that mediates the 3′ incision of the excision step [24]–[27]. Mutations in the *XPB* and *XPD* genes may also cause pure XP or trichothiodystrophy (TTD), a disorder that is characterized by sulphur-deficient hair, in association with a variable spectrum of abnormalities that usually include neurodevelopmental deficits. Mutations that cause XP preferentially afflict the NER activity of TFIIH, while TTD mutations destabilize the TFIIH complex causing exhaustion of TFIIH in specific cell types [27]. The occurrence of CS symptoms in association with specific XPB and XPD mutations point to functions beyond NER and basal transcription presumably linked to non-NER TCR activities akin to CSA and CSB [22], [27]–[31]. XPG mutations associated with XPCS have been proposed to destabilize the interaction between XPG and TFIIH, while mutations causing XP disrupt its endonuclease activity, further pointing to a non-NER activity underlying CS symptoms [25], [26], [32].

The presence of progressive juvenile or adult onset neurological abnormalities in XP-A patients has provided a strong hint that the NER pathway is important for neuronal function and maintenance [14], [19], [33]–[36]. The neurological symptoms are characterized by progressive sensory and motor deficits, as well as cognitive deterioration and emotional abnormalities, and are associated with widespread neuronal degeneration in multiple brain areas and the spinal cord [17]–[19]. XP-C patients (who are only deficient in GG-NER) do not develop overt neurological symptoms, indicating that the neurodegenerative changes follow from TC-NER or a combined GG-NER and TC-NER dysfunction. A dominant role of the TC-NER pathway in the nervous system is also suggested by the occurrence of XP-A-like progressive neurological abnormalities in CS patients. However, in CS patients neuropathological changes are primarily characterized by myelin abnormalities, while neurons and their axons seem relatively unaffected [14], [35], [37], [38]. An additional complicating factor is formed by patients carrying *CSA* or *CSB* mutations that develop UV-sensitive syndrome, a disorder that is characterized by the skin abnormalities of CS in the absence of other CS features. The lack of typical CS features in these patients has been linked to residual TCR activities required for repair of oxidative DNA lesions, while TC-NER of UV-induced DNA lesions was deficient [39]. In sum, the data from XP and CS patients indicate that combined deficiency of GG-NER and TC-NER as in XP-A patients predominantly afflicts neurons, while deficiencies of TCR predominantly cause myelin problems. However, the precise mechanisms underlying the differential cellular vulnerabilities in XP and CS nervous system are still poorly defined, in particular in CS where distinct degenerative mechanisms may operate in oligodendrocytes and neurons [7], [22].

Although previous studies have shown that mouse models for XP, CS, XP-CS and TTD reliably recapitulate the repair defect (i.e. GG-NER and/or TC-NER/TCR), UV-sensitivity and skin cancer predisposition associated with the corresponding NER syndromes (Table 1 and Table 2), this does not apply to the neurological features [40]. In particular, *Xpa^−/−^* mice fail to exhibit obvious neurological symptoms and neuropathological changes observed in human XP-A [41]–[44]. Likewise, *Csa^−/−^* and *Csb^−/−^* mouse models for CS, except for photoreceptor-loss, do not show overt neurological abnormalities [45], [46]. However, a detailed systematic neuropathological analysis is still lacking. In the present study we reexamined various NER and TCR mutant mouse models for neurodegenerative abnormalities to assess and dissect the contribution of the different repair systems in preventing neurodegeneration. To permit analysis of the direct contribution of DNA repair defects to neurological functioning, in the absence of pathology elsewhere in the body due to systemic DNA repair deficiency, we have generated a Cre-lox-based conditional *Xpa* mouse model that enables inactivation of the *Xpa* gene selectively in neurons. In particular, we used this novel mouse model to examine the effect of combined NER and TCR deficiency on neuronal survival.

**Table 1:** NER defect and sensitivity to genotoxins of embryonic fibroblasts isolated from NER–deficient mouse models.

Mouse Model	NER defect		Sensitivity to genotoxins			References
	GG-NER	TC-NER	UV	γ-irradiation	Paraquat	
***Xpa^−/−^***	+	+	+	−	−	[43], [47], [55]
***Xpc^−/−^***	+	−	+	−	−	[43], [47]
***Csa^−/−^***	−	+	+	−	−	[20], [46], [48], [53], [54], [55]
***Csb^−/−^***	−	+	+	+	+	[20], [45], [46], [48], [53], [54], [55]
***Xpd^XPCS^***	+/−	+	+	+	+	[28], [49]
***Xpd^TTD^***	+/−	+/−	+	+/−	N.D.	[50], [51], [53]

**Table 2:** Cancer susceptibility and principal features of NER–deficient mice.

Mouse Model	Cancer susceptibility			Median survival	Principal features	References
	untreated	UV-induced	DMBA-induced			
***Xpa^−/−^***	+/−	++	++	trend of reduced survival	normal weight, photosensitive skin and eyes, increased mutation frequencies, impaired spermatogenesis	[41], [43], [44], [52], [53], [56]
***Xpc^−/−^***	+/−	++	+	mildly reduced	normal weight, photosensitive skin and eyes, increased mutation frequencies	[43], [47], [52], [56]
***Csa^−/−^***	−	+	ND	ND	mildly reduced weight, photosensitive skin and eyes, retinal photoreceptor degeneration, increased vulnerability to γ-irradiation	[20], [46], [48], [54], [55]
***Csb^−/−^***	−	+	+	normal	mildly reduced weight, photosensitive skin and eyes, no changes in mutation frequency, retinal photoreceptor degeneration, increased vulnerability to γ-irradiation	[20], [45], [48], [54], [55]
***Xpd^XPCS^***	+	++	++	mildly reduced	mildly reduced weight, smaller testis	[28], [49]
***Xpd^TTD^***	−	+	+	mildly reduced	reduced weight, TTD-like brittle hair, mild osteoporosis, signs of accelerated aging, anaemia	[50], [51], [53]

## Results

### Sporadic p53 activation in astrocytes and neurons throughout the central nervous system of Cockayne syndrome mice

As a first step to study the role of NER and TCR in maintaining neuronal integrity, we have re-examined six previously reported mutant mouse models for the presence of neuropathological abnormalities. These mouse lines consisted of *Xpc^−/−^* mice, in which only GG-NER is completely inactive [43], [47]; *Xpa^−/−^* mice, in which both GG-NER and TC-NER are fully deficient [41], [43]; *Csa^−/−^* and *Csb^−/−^* mice, in which TC-NER and presumably the entire TCR pathway is abrogated, but which have proficient GG-NER [45], [46], [48]; *Xpd^XPCS^* mice (homozygous for the G602D XPCS mutation in the *Xpd* gene), which carry a partial GG-NER and a partial TCR defect [49]; and *Xpd^TTD^* mice carrying *Xpd* alleles with the R722W TTD mutation, that also have a partial GG-NER and TCR defect, in addition to TFIIH instability causing transcriptional insufficiency in terminally differentiated cells with consequent brittle hair and nails (Table 1 and Table 2) [50], [51]. Consistent with their respective NER-deficiencies the mutant mice manifest various degrees of increased susceptibility to UV- and 7,12-dimethylbenz[a]anthracene (DMBA)-induced skin carcinogenesis (Table 1 and Table 2). TCR-deficient mice to varying extent exhibit other symptoms like reduced growth, osteoporosis, photoreceptor loss, liver and kidney aging, and reduced lifespan (Table 2) [20], [28], [44]–[56].

**Figure 1:**
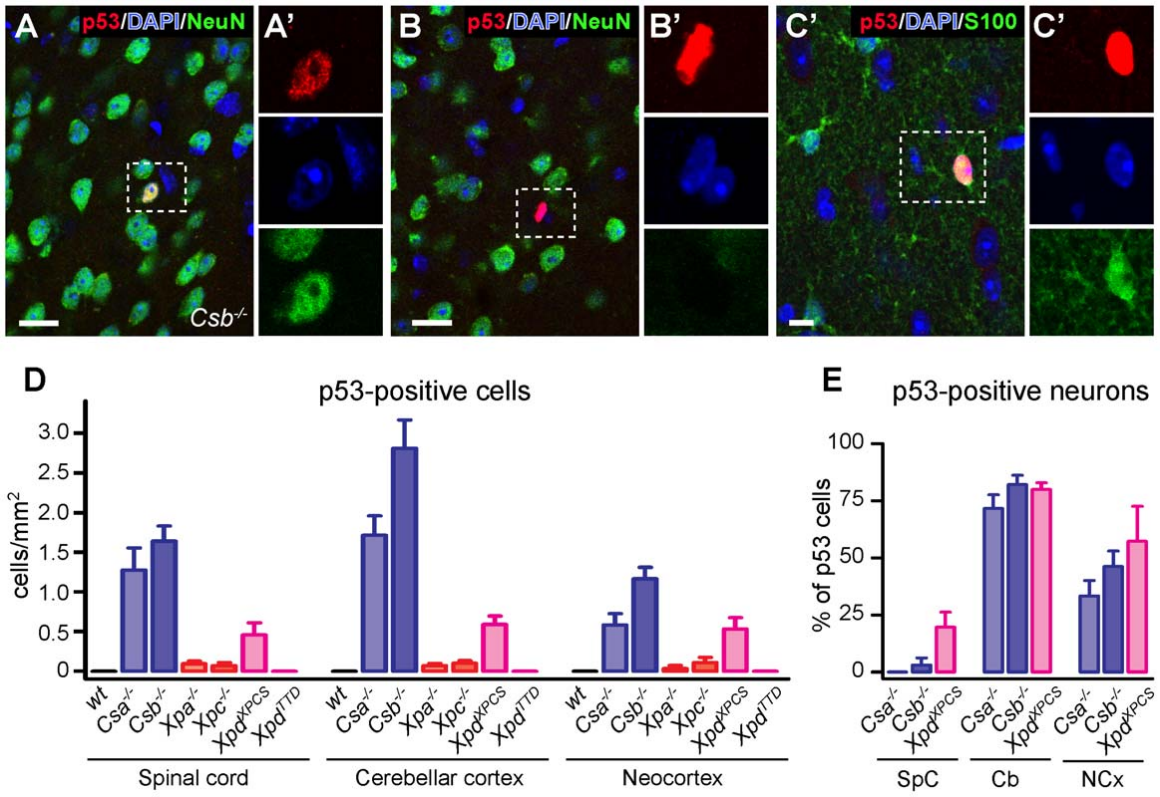
p53 expression in brain and spinal cord of adult NER– and TCR–deficient mice. A–C) Double-labeling confocal images showing p53-NeuN (A, B) and p53-S100 (C) double-labeled cells in neocortex of a 25 week old *Csb^−/−^* mouse. Scale bars: 25 µm (A, B) and 10 µm (C). D, E) Bar graphs showing the density of p53-labeled cells, and the relative amount of p53- neurons (i.e. percentage of NeuN-positive p53-labeled). Values are means ± SE of 3 or 4 mice, while value per mouse is based on analysis of 4 (neocortex [NCx], cerebellar cortex [Cb]) or 10 (lumbar L4 spinal cord [SpC]) sections.

Consistent with previous reports [43]–[50] analysis of thionin- and hematoxylin/eosin-stained brain sections revealed that the gross anatomy and histological organization of all central nervous system regions of aforementioned mutant mice were indistinguishable from wild type animals at 6 months of age, precluding overt neurodevelopmental deficits or neuronal degeneration. Also the central nervous system of 70–100 week old *Csb^−/−^* and *Xpa^−/−^* mice appeared normal. Therefore, to examine the possible occurrence of subtle abnormalities, we employed immunohistological approaches. First, to determine whether central nervous system cells of the NER-deficient mice experience genotoxic stress, we studied the expression of the transcription factor p53, which is activated by multiple types of DNA damage [57]. p53-immunoreactive cells were not detected in the central nervous system of wild-type and *Xpd^TTD^* mice. Instead, occasional cells with p53-immunoreactive nuclei were observed throughout the nervous system of *Csa^−/−^*, *Csb^−/−^* and *Xpd^XPCS^* mice, while even more sporadic p53 induction was visible in *Xpa^−/−^* and *Xpc^−/−^* animals (Figure 1). p53-staining was associated with neurons (NeuN-positive cells; Figure 1A), astrocytes (GFAP- or S100-positive cells; Figure 1C and Figure S1), and sometimes oligodendrocytes (APC-positive cells; Figure S1). The relative amount of glial versus neuronal p53-staining varied per brain region, and to some extent per mouse model: in cerebellar cortex the large majority of p53-positive cells consisted of neurons, in the neocortex p53-positive cells were neurons or non-neuronal cells in equal amounts, while in spinal cord and the brainstem reticular formation p53-immunoreactivity was predominantly or almost exclusively associated with glia cells (Figure 1E and Figure S1). p53-immunoreactive neurons generally showed normal nuclear morphologies, in contrast to most p53-immunoreactive glial cells. For instance, a subset of p53-positive astrocytes in spinal cord and the brainstem reticular formation showed nuclei with a DAPI-negative centre that was intensely p53-positive (Figure S1). Another nuclear abnormality of p53-positive glia consisted of a larger nuclear size (Figure S1). Also in *Xpa^−/−^* and *Xpc^−/−^* nervous systems p53-positive cells consisted of both neurons and astrocytes, but their frequency was too low to allow systematic analysis of the relative proportion of neuronal versus non-neuronal cells in different brain areas. Taken together the data indicate that mice with a complete (*Csa^−/−^*, *Csb^−/−^*) or severe partial (*Xpd^XPCS^*) TCR defect show nuclear p53 expression in sporadic neurons and glia throughout the nervous system, pointing to the occurrence of genotoxic stress.

### Microglia activation and astrocytosis in white matter regions of Cockayne syndrome *Csa^−/−^*, *Csb^−/−^*, and *Xpd^XPCS^* mice

**Figure 2:**
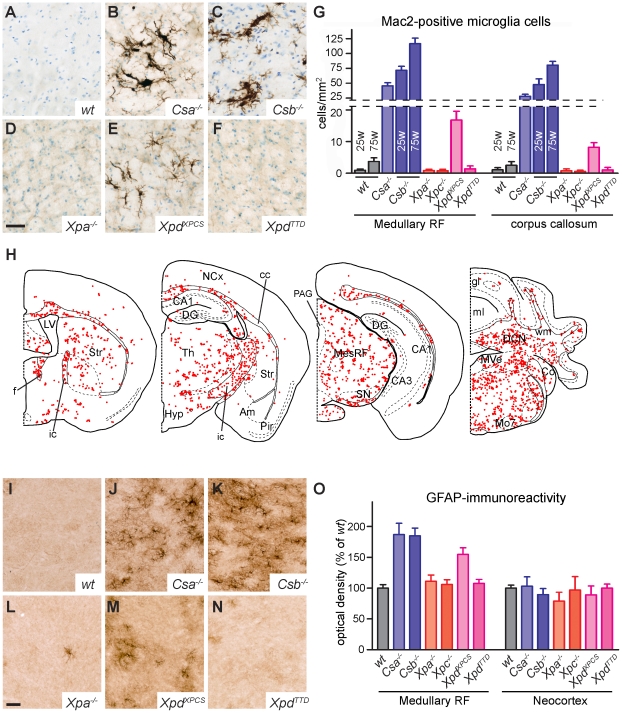
TCR–deficient mice show microglia and astrocyte activation in the white matter. A–F) Mac2-immunostaining of activated microglia in the medullary reticular formation of 25 week old wild-type and NER-deficient mice showing the presence of Mac2-positive microglia cells in the reticular formation of *Csa^−/−^* (B), *Csb^−/−^* (C) and *Xpd^XPCS^* (E) mice. G) Bar graph showing the density of Mac2-labeled cells in the medullary reticular formation and corpus callosum of NER-mutant mice. Values are means ± SE of 3 or 4 mice, while values per mouse are based on analysis of 4 sections. H) Plot of Mac2-positive cells in selected transverse brain sections of 25 week old *Csb^−/−^* mouse illustrating the widespread distribution of activated microglial cells in regions containing myelinated axons such as the corpus callosum (cc), the internal capsule (ic), the fornix (f), the cerebellar white matter (wm), the mesencephalic (MesRF) and medullary reticular formation. No or a low number of Mac2- immunoreactive cells occur in grey matter regions, such as the Neocortex (NCx), the hippocampal subfields (DG [dentate gyrus], CA1 and CA3), amygdala (Am), piriform cortex (Pir), the cerebellar cortical granular (gl) and molecular layers (ml), and the medial (MVe) and lateral vestibular (LVe) nuclei. I–O) Photomicrographs and optical density measurements (O) of GFAP-immunoperoxidase staining in the medullary reticular formation of NER-deficient mice showing increased staining in *Csa^−/−^*, *Csb^−/−^* and *Xpd^XPCS^* mice as compared to the other genotypes. Optical densities were determined from TIFF files using MetaMorph 4.6 image analysis software. Values are means ± SE of 3 mice, while values per mouse are from 3 sections. To minimize variability resulting from the staining procedure all sections used for the graph were stained in a single session using the same reagents. Scale bars: 50 µm (D, L).

To further investigate the presence of degenerative changes in the nervous system of NER-deficient mice we examined microglia cells which proliferate and acquire activated morphologies in conditions of neuronal and glial damage [58], [59]. Immunostaining for Iba-1, a marker of all microglia cells and Mac2 (also known as galectin-3), a protein selectively expressed by activated phagocytosing microglia [58], did not, or only sporadically, reveal activated microglia cells in the nervous system of wild-type, *Xpa^−/−^*, *Xpc^−/−^* and *Xpd^TTD^* mice (Figure 2 and Figure S2). In contrast, prominent levels of activated microglia were present throughout the nervous system of *Csa^−/−^*, *Csb^−/−^* mice and, to a somewhat lesser extent, *Xpd^XPCS^* mice (Figure 2, Figures S2 and S3). Typically, Mac2-positive microglia cells occurred in small clusters in areas with myelinated fibers. Thus, in the forebrain Mac2-positive microglial cells were concentrated in the corpus callosum, the anterior commissure, the capsula interna and the fornix, while in the caudal brain high levels of activated microglia occurred in the cerebellar white matter, throughout the reticular formation and in fiber tracts in the brainstem (Figure 2, Figure S2 and Figure S3). We performed a more in-dept analysis of the time of onset and course of these features in *Csb^−/−^* mice at different ages. Prominent levels of Mac2-positive cells were already present before 10 weeks of age (not shown), and the density of Mac2-positive cells did not show a distinct increase with age (Figure 2G).

To examine whether microglia activation is paralleled by changes in astrocytes, we also stained for glial fibrillary acidic protein (GFAP), an astrocytic protein up-regulated under conditions of neuronal injury. Increased GFAP staining was observed in the nervous system of mouse mutants that also showed microglial cell activation, i.e. *Csa^−/−^*, *Csb^−/−^* and *Xpd^XPCS^* mice (Figure 2I–2O). Increased GFAP staining was most prominent in the brainstem reticular formation and spinal cord (Figure S4). No obvious changes in GFAP staining were noted in some white matter areas such as the corpus callosum and the capsula interna of *Csa^−/−^*, *Csb^−/−^* and *Xpd^XPCS^* mice, which may be explained by relatively higher baseline GFAP-immunoreactivity in these areas in wild-type mice. To further examine astrocytic changes in white matter areas, we examined the expression of Hsp25 (also known as Hsp27 or Hspb1), a small heat shock protein that is expressed at high levels in a subset of astrocytes in conditions of injury [60]. Indeed *Csa^−/−^*, *Csb^−/−^* and *Xpd^XPCS^*, but not *Xpa^−/−^*, *Xpc^−/−^* and *Xpd^TTD^* nervous systems showed the appearance of intensely Hsp25-immunoreactive astrocytes in multiple regions including the brainstem reticular formation, spinal cord, the white matter of cerebellum and the corpus callosum forebrain (Figure S4).

**Figure 3:**
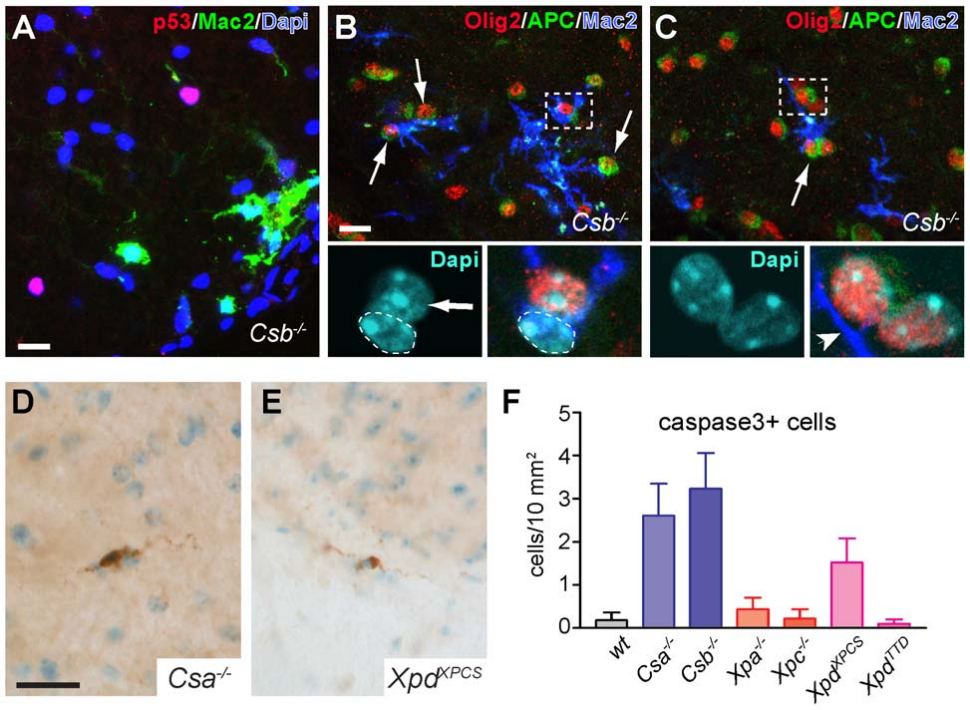
Mac2-positive microglia cells enwrap oligodendrocytes in CS mice. A) Double-labeling confocal images of p53 and Mac2 in DAPI-counterstained spinal cord section of *Csb^−/−^* mouse showing that Mac2-immunoreactive microglia cells do not colocalize with p53-labeled cells. B, C) Triple-labeling confocal images of Mac2 and the oligodendrocyte markers Olig2 and APC showing Mac2-immunoreactive microglia cells that closely embrace mature oligodendrocytes labeled by Olig2 and APC. The dashed line in (B) outlines the nucleus of the Mac2-positive microglia cell. Arrows in B and C indicate oligodendrocytes contacted by Mac2-positive microglia cells. D–F) Photomicrographs and bar graph of active caspase 3 immunoreactive cells in spinal cord of 25 week old *Csa^−/−^*, *Csb^−/−^* and *Xpd^XPCS^* mice. Values are means ± SE of 3 or 4 mice, while value per mouse is based on analysis of 10 cervical spinal cord sections. Scale bars: 20 µm.

The above data indicate that *Csa^−/−^*, *Csb^−/−^* and *Xpd^XPCS^* mice show microglia and astrocyte activation in multiple central nervous system areas, indicative of the occurrence of cellular degeneration or another detrimental process. As these mutant mice also displayed p53-immunoreactive cells, we performed double labeling of p53 with Mac2 to determine whether p53-immunoreactive cells are contacted by phagocytosing microglia cells. However, Mac2-positive microglia cells were never found in the vicinity of p53 cells (Figure 3A). Instead, Mac2-positive microglia were frequently in close proximity of oligodendrocytes (Olig2 and APC-positive; Figure 3B, 3C), that otherwise showed a healthy appearance with normal DAPI-stained nuclei. These data indicate that glial abnormalities may be associated with subtle alterations in oligodendrocytes. To examine whether the presence of activated microglia was associated with myelin abnormalities, we performed double labeling of neurofilament-H and myelin basic protein to outline axons and their myelin sheets. No differences in myelin basic protein staining were observed in spinal white matter and corpus callosum of wild-type and *Csb^−/−^* mice (not shown).

Finally, to determine whether *Csa^−/−^*, *Csb^−/−^* and *Xpd^XPCS^* mice show increased levels of cell death of oligodendrocytes or other cells, we stained for active caspase 3, which is a final executioner caspase associated with multiple cell death pathways [61]. Very sporadically caspase 3 immunoreactive cells were observed in the nervous system of all mutant mouse models; all positive cells showing morphologies compatible with glial cells (Figure 3D, 3E). Quantitative analysis in spinal cord indicated that the number of active caspase 3-positive cells, although still very low, was higher in *Csa^−/−^*, *Csb^−/−^* and *Xpd^XPCS^* mice as compared to the other genotypes (Figure 3F). It was not possible to determine whether the active caspase 3 cells represented oligodendrocytes or astrocytes because they did not stain for cellular markers such as GFAP, S100, APC ad NeuN. Consistent with active caspase 3 staining, a silver degeneration procedure, that outlines degenerating neurons and their processes, indicated that none of the NER mice showed detectable levels of neuronal degeneration at 26 weeks of age. Taken together the data show that *Csa^−/−^*, *Csb^−/−^* and *Xpd^XPCS^* mice develop prominent microglia cell activation as well as astrocytic changes that may be mostly triggered by subtle oligodendrocyte abnormalities.

### Neuron-specific inactivation of *Xpa* causes age-dependent neuronal loss in *Csb^−/−^* mice

The NER mouse models investigated above exhibited either no detectable neuronal abnormalities or evidence for only subtle neuronal dysfunction and degeneration, indicating that inactivation of NER or TCR pathways by itself is not sufficient to significantly affect long-term survival of neurons in mice. Previous studies disclosed synergistic deleterious effects of intercrossing XP (*Xpa^−/−^* or *Xpc^−/−^*) with CS (*Csa^−/−^*, *Csb^−/−^*, *Xpd^XPCS^*) mice, resulting in double mutants with very short life span and dramatic progeroid features [49], [62]–[64]. This raises the possibility that neuronal degeneration may be achieved by inactivation of multiple NER components afflicting both NER and TCR pathways. However, the very short lifespan as well as the serious systemic abnormalities of the double mutant mice precludes systematic analysis of neuronal degeneration, which could also be an indirect consequence of impaired function of other organs and systems. To address this issue, we generated a Cre-lox-based conditional *Xpa* mouse model that enables selective inactivation of the *Xpa* gene in postnatal neurons of CS mouse lines and hence to study the effect of combined TCR and NER inactivation in neurons of adult mice that do not suffer from other severe deficits.

**Figure 4:**
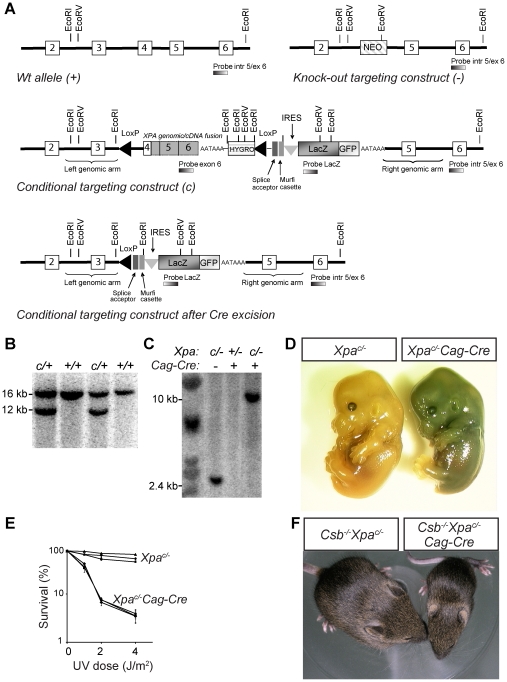
Generation of conditional *Xpa^c/−^* mouse with a floxed genomic/cDNA fusion construct. A) Schematic representation of the wild-type mouse *Xpa* locus (exon 2 to 6), the targeting construct, the conditional *Xpa* allele, and the conditional *Xpa* allele following Cre-recombinase-mediated inactivation. In addition the knock out allele with a neomycin (NEO) marker cassette, as present in the non-conditional *Xpa* mouse model is shown [41]. In the conditional construct, exon 4 was fused in frame to the mouse *Xpa* cDNA (containing the remaining coding sequence and including a synthetic polyA sequence), followed by a PGK promoter-driven hygromycin (HYGRO) selectable marker gene. LoxP sites were introduced in intron 3 and downstream of the hygromycin marker to allow Cre-mediated excision of the cDNA and hygromycin marker gene, yielding an *Xpa* allele lacking exon 4, known to act as a null allele. A LacZ-GFP fusion marker gene, preceded by a splice acceptor (SA) and an internal ribosomal entry (IRES) was included to allow visualization of inactivation of the conditional *Xpa* allele. Open and gray boxes represent exon and cDNA sequences, respectively. The splice acceptor-Murfi cassette ensures proper splicing and translational stops in all frames respectively when the *Xpa* gene is knocked out. B) Southern analysis of wild-type ES cell DNA transfected with the conditional *Xpa* construct. DNA was digested with EcoRI and hybridized with an intron 5/exon 6 probe, external to the targeting construct. Wild-type and targeted alleles gave fragments of 16 and 12 kb, respectively. C) Southern blot analysis of DNA from E13.5 *Xpa* ^c/−^ and *Xpa* ^c/−^/*Cag-Cre* embryos. DNA was digested with EcoRV and hybridized with a LacZ probe. Intact and Cre-inactivated conditional *Xpa* alleles are represented by 2.4 and 10 kb fragments, respectively. D) LacZ staining of E13.5 *Xpa* ^c/−^ and *Xpa* ^c/−^/*Cag-Cre* embryos, indicating inactivation of the conditional *Xpa* allele in *Xpa* ^c/−^/*Cag-Cre* embryos only. E) Survival of wild-type, *Xpa* ^c/−^ and *Xpa* ^c/−^/*Cag-Cre* MEFs, exposed to increasing doses of UV-C light. F) Photograph of 16-day old *Csb^−/−^*/*Xpa* ^c/−^ and *Csb^−/−^*/*Xpa* ^c/−^/*Cag-Cre* littermates, the latter showing severely reduced size.

To establish a conditional *Xpa* knockout mouse model, we generated a targeting construct in which exon 4 of the *Xpa* gene is fused in frame to the mouse *Xpa* cDNA containing the remaining coding sequence and including a synthetic polyA sequence, followed by a PGK promoter-driven hygromycin selectable marker gene, and a LacZ-GFP fusion gene (Figure 4A). The splice acceptor-Murfi cassette ensures proper splicing and translational stops in all frames respectively when the *Xpa* gene is knocked out (Figure 4A). The functionality of this conditional genomic-cDNA fusion allele was tested in UV-hypersensitive *Xpa^−/−^* ES cells (Figure S5). These experiments showed that the *Xpa^c^* conditional allele fully averted the UV-hypersensitivity of *Xpa^−/−^* ES cells (Figure S5). Next *Xpa^c/+^* ES cells, obtained by transfection of IB10 ES cells (Figure 4B), were used for blastocyst injections and subsequent generation of *Xpa^c/+^* mice. To determine whether Cre recombinase was capable of excising the floxed *Xpa* sequence *in vivo*, we generated *Xpa^c/−^/Cag-Cre* mice by crossing *Xpa^c/+^* mice with *Xpa^+/−^* mice carrying a *Cag*-promotor driven *Cre* transgene (*Cag-Cre*), which drives Cre recombinase expression immediately after conception [65]. Southern blot analysis showed Cre-recombinase excision of the floxed sequence in *Xpa^c/−^/Cag-Cre* embryos at ∼100% efficiency (Figure 4C). Consistent with ubiquitous recombination, *Xpa^c/−^/Cag-Cre* embryos stained blue upon X-gal staining due to LacZ expression, while *Xpa^c/−^* embryos remained unstained (Figure 4D). *Xpa^c/−^/Cag-Cre* mouse embryonic fibroblasts (MEFs) like *Xpa^−/−^* MEFs [41] showed severe UV-hypersensitivity, while *Xpa^c/−^* MEFs show wild-type UV-resistance, consistent with Cre-dependent inactivation of the conditional allele (Figure 4E). Next, we crossed *Csb^−/−^/Xpa^c/+^* and *Csb^−/−^/Xpa^+/−^/Cag-Cre* mice to obtain *Csb^−/−^/Xpa^c/−^* and *Csb^−/−^/Xpa^c/−^/Cag-Cre* mice. In line with the phenotype of *Csb^−/−^/Xpa^−/−^* mice [63], *Csb^−/−^/Xpa^c/−^Cag-Cre* pups displayed severe postnatal growth deficits, cachexia, disturbed gait, and death before weaning, while *Csb^−/−^/Xpa^c/−^* littermates did not develop overt pathology (Figure 4F). Taken together, these data demonstrate that we have generated a valid conditional *Xpa* mouse model that enables us to study the *Csb^−/−^/Xpa^−/−^* phenotype in a cell or tissue-specific manner.

**Figure 5:**
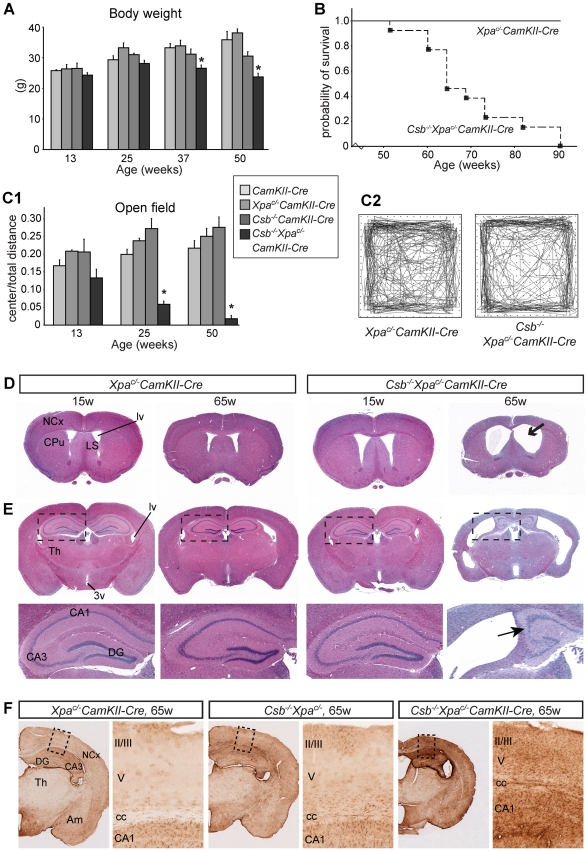
Reduced lifespan, age-dependent behavioral abnormalities, and brain atrophy in forebrain neuron-specific knockout of *Xpa* in *Csb^−/−^* mice. A) Mean body weight of *CamKIIα-Cre*, *Csb^−/−^CamKIIα-Cre*, *Xpa* ^c/−^ *CamKIIα-Cre* and *Csb^−/−^*/*Xpa* ^c/−^/*CamKIIα-Cre* mice. Values are means ± SE (n = 8 to 13/group). Note the loss of body weight in *Csb^m/m^*/*Xpa* ^c/−^/*CamKIIα-Cre* mice after the age of 25 weeks. * represent P<0.05, as compared to other groups of the same age (one-way Anova with Tukey's post-test). B) Survival curve of *Csb^−/−^*/*Xpa* ^c/−^/*CamKIIα-Cre* mice (n = 13) as compared to wild type and single mutant littermates (combined n = 13). C) Representative examples of open field plots (C2) and quantification of the distance moved in the center as compared to total distance moved (C1), showing reduced ambulatory behavior of *Csb^−/−^*/*Xpa* ^c/−^/*CamKIIα-Cre* mice (n = 6) in the center of the field. Note that the center/total distance ratio is already reduced in 3 month-old *Csb^m/m^*/*Xpa* ^c/−^/*CamKIIα-Cre* mice, and decreases upon further aging. * in panel C, P<0.05, compared to other groups of the same age (one-way Anova with Tukey's post test). D, E) Coronal 4 µm thick, haematoxylin/eosin-stained paraffin sections of 15 and 65 week old *Xpa* ^c/−^/*CamKIIα-Cre* and *Csb^−/−^*/*Xpa* ^c/−^/*CamKIIα-Cre* mouse brains; sections grossly correspond to sections 0.5 to 1 mm anterior to the bregma (D) and 1.5 to 2 mm posterior to the bregma (E) as depicted in the mouse brain atlas of Paxinos and Franklin [85]. 65 week old *Csb^−/−^*/*Xpa* ^c/−^/*CamKIIα-Cre* show dilated ventricles, and atrophy of the neocortex (NCx), hippocampus, the caudatus-putamen (CPu) and the septum. CA3, CA3 hippocampal subfields; DG, dentate gyrus; Th, thalamus; LS, lateral septum; lv, lateral ventricle. F) GFAP-immunoperoxidase staining in coronal brain sections of 65 week-old *Xpa* ^c/−^ *CamKIIα-Cre*, *Csb^−/−^* and *Csb^−/−^Xpa* ^c/−^ *CamKIIα-Cre* shows increased GFAP staining in the neocortex (NCx), hippocampus and amygdala of *Csb^−/−^Xpa* ^c/−^ *CamKIIα-Cre* mice. GFAP staining in the thalamus (Th) was the same as in *Xpa* ^c/−^ *CamKIIα-Cre* and *Csb^−/−^* mice.

To study the effect of *Xpa* inactivation in the absence of Csb, specifically in postnatal neurons, we crossed *Csb^−/−^/Xpa^c/−^* mice with a calcium/calmodulin-dependent protein kinase IIα (CamKIIα) Cre transgenic line that expresses Cre-recombinase selectively in postnatal neurons throughout the forebrain [66], [67]. Forebrain-specific recombination was confirmed by PCR and analysis of LacZ expression. *Csb^−/−^/Xpa^c/−^/CamKIIα-Cre* mice grew into young adulthood without any noticeable phenotype, showed a normal body weight and appearance at the age of 6 months, but from the age of 9–12 months became smaller and exhibited reduced weight as compared to littermates with other genotypes, i.e. *CamKIIα-Cre*, *Csb^−/−^CamKIIα-Cre*, *Xpa^c/−^CamKIIα-Cre* and *Csb^−/−^/Xpa^c/−^* littermates (Figure 5A, Figure S6). In addition, from 9–12 months of age, *Csb^−/−^/Xpa^c/−^/CamKII-Cre* mice started to display seizure behavior, characterized by episodes of immobility. Subsequently, *Csb^−/−^/Xpa^c/−^/CamKII-Cre* mice became moribund, all animals dying prematurely between the age of 12 and 22 months (Figure 5B), while animals from littermates with other genotypes survived up to 24 months (the oldest age examined). Analysis of locomotor behavior using the accelerating rotarod assay demonstrated that *Csb^−/−^/Xpa^c/−^/CamKIIα-Cre* mice performed within the normal range at the age of 6 months, but showed reduced performance at 12 months (Figure S6). For further analysis of behavioral abnormalities, we used an open-field exploratory test. This test revealed that *Csb^−/−^/Xpa^c/−^/CamKIIα-Cre* mice avoided exploration of the central part of the open field, which is considered a measure of anxiety-related behavior [68]. Total movement time and distance were the same as for the other groups ruling out impaired mobility as explanation for the difference in the test. The ratio of the ambulatory activity in the center and the total walking distance was already reduced at 3 months of age, and further declined at 6 and 12 months of age (Figure 5C).

Macroscopic examination of the brain of *Csb^−/−^/Xpa^c/−^/CamKIIα-Cre* mice revealed no obvious changes at 3 months, mild atrophy of the cortex at 6 months, and severe atrophy of the cortex at older age (Figure S6). The sizes of olfactory bulbs, cerebellum and spinal cord were the same as in other groups. Analysis of coronal sections of 12–16 month-old *Csb^−/−^/Xpa^c/−^/CamKIIα-Cre* brains showed a large reduction in cortical thickness, atrophy of other telencephalic areas (i.e. hippocampus, caudatus-putamen and septum), and dramatically enlarged lateral ventricles (Figure 5D, 5E). No abnormalities were observed in non-telencephalic areas, consistent with specific inactivation of the conditional *Xpa* allele in forebrain neurons. Atrophy of telencephalic areas was paralleled by a marked increase in GFAP immunoreactivity, while GFAP staining in other brain areas was the same as in *Csb^−/−^/Xpa^c/−^* and *Csb^−/−^* mice (Figure 5F). Atrophied brain areas also showed loss of the neuronal somato-dendritic marker microtubule-associated protein 2 (MAP2), in particular in the hippocampal CA1 region, indicative of neuronal degeneration (Figure S7).

**Figure 6:**
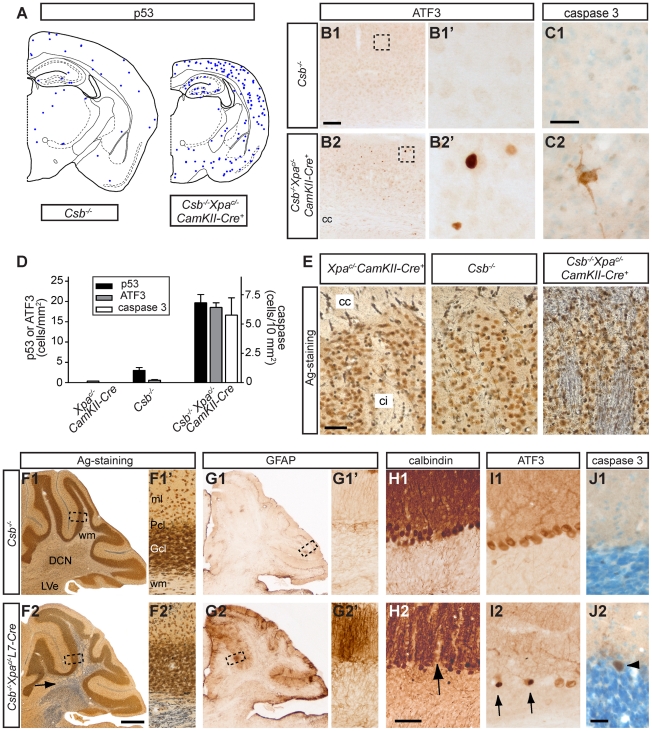
Neurodegenerative changes in forebrain neuron-specific and Purkinje cell-specific knockout of *Xpa* in *Csb^−/−^* mice. A–D) Neurolucida plot (A), representative photomicrographs (B, C) and bar graph (D) of p53 (A, D), ATF3 (B, D) and active caspase 3 (C, D) staining in the cortex of 65 week old *Csb^−/−^* and *Csb^−/−^Xpa* ^c/−^ *CamKIIα-Cre* mice showing an increase in the number of p53- and ATF3 immunoreactive cells and the appearance of caspase 3 labeled neurons in the cortex of *Csb^−/−^Xpa* ^c/−^ *CamKIIα-Cre* mice. Values in bar graph in d represent means ± SE from 3 mice per group. E) Silver degeneration staining in the caudatus-putamen shows a high level of argyrophilic fibers in the capsula interna (ci) and corpus callosum (cc) of 16 month-old *Csb^−/−^Xpa* ^c/−^ *CamKIIα-Cre* mice while no stained fibers occurred in these fiber tracts in wild-type (not shown), *Csb^−/−^* and *Xpa* ^c/−^ *CamKIIα-Cre* mice. F–J) Photomicrographs of sagittal cerebellar sections of 25-week old *Csb^−/−^* (F1–J1) and *Csb^−/−^Xpa* ^c/−^ *L7-Cre* mice (F2–J2). F) Silver degeneration staining shows a high level of argyrophilic fibers in the deep cerebellar nuclei (DCN) and the white matter (wm) of *Csb^−/−^Xpa* ^c/−^ *L7-Cre* cerebellum (arrow in F2). G) GFAP-immunoperoxidase staining shows increased GFAP staining in the molecular (ml) and Purkinje cell (Pcl) layers *Csb^−/−^Xpa* ^c/−^ *L7-Cre* cerebellar cortex, while no change occur in the granule cell layer (Gcl). H) Calbindin-immunoperoxidase staining reveals calbindin-negative regions in the molecular layer (arrow in H2) of *Csb^−/−^Xpa* ^c/−^ *L7-Cre* cerebellar cortex. I) A number of Purkinje cells showed strong nuclear ATF3 staining (arrow in I2). Note that the ATF3 antibody also outlines Purkinje cells because of non-specific cytoplasmatic staining of these cells. J) Active caspase 3 antibody stains sporadic Purkinje cells in *Csb^−/−^Xpa* ^c/−^ *L7-Cre* cerebellar cortex (arrow head in J2). Calibration Bars: Bars: 500 µm (F2), 100 (B1), 50 µm (E, H2), 25 µm (C1, J2).

Staining for p53 revealed a prominent increase in the number of p53 immunoreactive neurons in the forebrain of *Csb^−/−^/Xpa^c/−^/CamKIIα-Cre* mice as compared to *Csb^−/−^* mice and other genotypes that showed essentially no p53 immunoreactive cells (Figure 6A, 6D). In addition, *Csb^−/−^/Xpa^c/−^/CamKIIα-Cre* forebrain exhibited a strong increase in neurons expressing ATF3 (Figure 6B, 6D), a stress-inducible transcription factor that is induced following genotoxic stress via p53-dependent and -independent pathways [69], [70]. Finally, direct evidence for neuronal degeneration was obtained by staining for active caspase 3 and by using a silver staining procedure: active caspase 3 staining revealed intensely stained neuronal profiles (Figure 6C, 6D). Similarly, the silver degeneration staining method outlined infrequent argyrophylic neuronal profiles, reflecting neurons that are in the process of dying. In addition, the silver staining uncovered high levels of argyrophilic axonal degeneration in the corpus callosum, the fimbria-fornix, the anterior commissure, and the cortifugal fiber bundles coursing in the capsula interna, the cerebral peduncle and the pyramidal tract (Figure 6E), which is consistent with the selective occurrence of neuronal degeneration in forebrain neurons. Together these data indicate that *Csb^−/−^/Xpa^c/−^/CamKII-Cre* mice display chronic neuronal degeneration that in the long term has resulted in severe neuronal loss and atrophy of the forebrain regions.

The distribution of degenerative changes in *Csb^−/−^/Xpa^c/−^/CamKII-Cre* mice is consistent with the specific inactivation of *Xpa* in forebrain neurons induced by CamKII-promotor driven Cre-recombinase expression [66], [67], and highlights the vulnerability of Csb-deficient forebrain neurons to loss of Xpa function. To determine the effect of *Xpa* inactivation in other neuronal populations of the *Csb^−/−^* brain, we crossed *Csb^−/−^Xpa^c/−^* mice with a postnatal Purkinje cell specific Cre (*L7-Cre*) transgenic line [71] to obtain *Csb^−/−^/Xpa^c/−^/L7-Cre* mice. Analysis of motor behavior with accelerating rotarod revealed no or very mild motor abnormalities in *Csb^−/−^/Xpa^c/−^/L7-Cre* mice at the age of 3 and 6 months (the oldest age examined). However, neuropathological analysis disclosed multiple signs of selective Purkinje cell degeneration resembling pathological changes in forebrain neurons of *Csb^−/−^/Xpa^c/−^/CamKII-Cre* mice (Figure 6). Abnormalities included the presence of argyrophilic axonal degeneration, specifically in the cerebellar white matter and cerebellar nuclei, i.e. the areas that contain Purkinje cell axons (Figure 6F), and sporadic argyrophilic debris in the molecular and Purkinje cell layer, while no argyrophilic changes occurred in other brain areas. In addition, the Purkinje and molecular layers also showed a strong increase in GFAP-immunoreactivity (Figure 6G), while staining for calbindin, a protein that in the cerebellum is selectively expressed in Purkinje cells, revealed calbindin-negative regions in the molecular layer, indicative of loss of Purkinje cells (Figure 6H). Furthermore, a subset of Purkinje cells (with morphologies varying from relatively normal to severely atrophic cells) displayed strong nuclear ATF3 staining (Figure 6I), which was distinct from the non-specific cytoplasmic staining of Purkinje cells produced by the ATF3 antibody. Nuclear ATF3 staining was not observed in Purkinje cells (nor other cerebellar neurons) of wild-type, *Csb^−/−^*, *Csb^−/−^/Xpa^c/+^/L7Cre*, *Csb^−/−^/Xpa^c/−^* and *Xpa^c/−^/L7-Cre*, as well as forebrain-specific *Csb^−/−^/Xpa^c/−^/CamKII-Cre* mice. Quantification of ATF3-immunoreactive Purkinje cells in mid-sagittal sections of 6 month-old *Csb^−/−^/Xpa^c/−^/L7-Cre* mice (n = 3) indicated that 1.3±0.6% (Mean ± SE) of Purkinje cells were ATF3-positive. Finally, staining for active caspase 3 revealed infrequent (<1 in 5000) positive Purkinje cells (Figure 6J). In conclusion, the data obtained with *Csb^−/−^/Xpa^c/−^/L7-Cre* mice further demonstrate that the addition of an *Xpa* defect to Csb-deficient neurons results in pronounced neuronal degeneration.

## Discussion

To determine the importance of the NER and TCR DNA repair pathways for neuronal integrity and survival, we first conducted a comparative analysis of neuropathological abnormalities in six mouse models for the human syndromes XP, CS, XPCS and TTD. The major finding of this analysis is that CS-like/TCR-deficient *Csa^−/−^*, *Csb^−/−^* mice and, to a lesser extent, *Xpd^XPCS^* mice, develop a characteristic set of mild degenerative changes that was predominantly characterized by microglia activation in regions with myelinated axons, in the absence of obvious signs of axonal degeneration. This microglia activation did not occur in the XP-like GG-NER-defective *Xpc^−/−^* and total NER-defective *Xpa^−/−^* mutant animals, nor in the TTD-like *Xpd^TTD^* mouse. In view of very limited signs of neuronal degeneration in NER- and TCR-deficient mice, respectively, we also investigated the effect of combined NER- and TCR-deficiency on neuronal survival. For this purpose we generated a Cre-lox based conditional *Xpa* knockout mouse that was crossed with neuron-specific Cre lines and TCR-deficient *Csb^−/−^* mice. These experiments showed that combining NER and TCR-defects in neurons causes progressive neuronal degeneration and disclose a functional overlap as well as functional complementarity of the NER and TCR repair pathways.

### Identification of a CS–like neuropathological phenotype in TCR–deficient mouse mutants

The abnormalities identified in the CS (*Csa^−/−^* and *Csb^−/−^*), and XPCS (*Xpd^XPCS^*) mice consisted of 1) the presence of activated phagocytosing microglia cells in regions containing myelinated axons such as the corpus callosum, the brainstem reticular formation and the spinal cord; and 2) sporadic cells with intense p53-immunoreactive nuclei. Microglia activation was frequently accompanied by signs of astrocytosis indicative of a central nervous tissue injury response. We did not find an association between microglia activation and p53-positive cells, and neither was microglia activation associated with detectable axonal degeneration. However, activated microglia cells were often in close contact with oligodendrocytes. These data indicate that microglia activation follows from oligodendrocyte or myelin abnormalities. Previous electron microscopic analysis did not reveal abnormalities in the morphology and thickness of myelin sheets in *Csb^−/−^* mice [45], and in the current study, apart from evidence suggesting a minor increase in apoptosis of oligodendrocytes, we did not identify other overt oligodendrocytic abnormalities. Hence, the precise cellular abnormality that triggers microglia activation in myelinated regions of *Csa^−/−^*, *Csb^−/−^*, and *Xpd^XPCS^* mice remains to be determined. Importantly, however, the presence of activated microglia is consistent with the notion that irregular patchy myelination with minimal axonal degeneration is a dominant neuropathological hallmark of CS [14], [22], [35], [37], [72]. Hence, our findings together with human neuropathological data strongly indicate that oligodendrocyte abnormalities are a prime defect in CS. We also show that *Xpd^TTD^* mice, unlike *Xpd^XPCS^* mice, do not develop microglia activation in myelinated areas. This is in line with the notion that myelin abnormalities in TTD patients and *Xpd^TTD^* mice result from developmental deficits and arise via different mechanisms than in CS patients [14], [22]. Our data further illustrate that specific point mutations in the *Xpd* gene result in different cellular deficits and associated pathologies in the mouse, mimicking the different pathologies in patients [27], [31], [49].

The second abnormality that we identified in the CS (*Csa^−/−^* and *Csb^−/−^*) and the XPCS (*Xpd^XPCS^*) mouse nervous systems consisted of sporadically distributed p53-immunoreactive neurons and astrocytes, and, albeit very infrequent, oligodendrocytes. p53-immunoreactive cells occurred in all brain areas, but the proportion of neuronal versus glial p53-immunoreactive cells varied among brain areas. Thus, in cortex and cerebellum a large proportion of p53-positive cells are neurons while in the brain stem and spinal cord the far majority, if not all, p53-positive cells are glial cells. The expression of p53, which is known to be activated by multiple types of DNA damage and which mediates neuronal degeneration [57], provides indirect evidence for the occurrence of genotoxic stress, which can be explained by cumulative DNA damage resulting from compromised DNA repair. Interestingly, a subset of p53-positive astrocytes showed abnormal nuclear morphologies, which is compatible with reports of astrocytic nuclear abnormalities in CS patients [38], [72], [73], and further indicate that astrocytes are vulnerable to loss of TCR function.

We did not observe abnormal nuclear morphology in p53-immunoreactive neurons, nor did we obtain direct evidence for ongoing neuronal death using two neuropathological markers for dying neurons, i.e. active caspase 3 immunoreactivity, and silver degeneration staining. However, the process of death and removal of individual neurons may occur within a few hours, making the *in vivo* detection of asynchronous sporadically distributed cell death challenging [74], [75]. Hence, our methods do not exclude the possibility of a low frequency of ongoing neuronal degeneration. The lack of an obvious neurodegenerative phenotype in the CS mouse models is compatible with the neuropathology of CS patients indicating relatively modest neuronal degeneration in most brain areas [35], [38], [72], [73]. Interestingly, cerebellar granule cells, which are among the most severely affected populations of neurons in CS patients [76], most frequently showed p53 immunoreactivity in the CS mice, indicative of a differential vulnerability of cerebellar granule cells to loss of TCR function in both CS patients and mouse models. Furthermore, p53-immunoreactive granule cells have been demonstrated in autopsy cases of CS [76].

Together our data indicate that *Csa^−/−^*, *Csb^−/−^*, and *Xpd^XPCS^* mice reproduce the major aspects of CS neuropathology, albeit in a mild form, which may explain the absence of macroscopic neuropathological and obvious neurological deficits associated with CS. In this context it would be interesting to know whether patients with UV-sensitivity syndrome (UVSS), also carrying mutations in *CSA* and *CSB* genes, develop the same mild abnormalities. The presence of activated microglia in UVSS patients would support the notion of a continuum of CS phenotypes ranging from CS type II to UVSS [16] as also suggested by a CS patient with a CSB null mutation displaying adult-onset neurological symptoms [37]. As the pathologies of *Csa^−/−^*, *Csb^−/−^*, and *Xpd^XPCS^* mice are relatively similar, our data also indicate that the CS neurodegenerative features can not be explained by molecular mechanisms that do not include all three proteins. Furthermore, the data indicate that the CS neurodegenerative changes result from deficits in a shared non-NER activity of these proteins as *Xpa^−/−^* mice with complete loss of GG-NER and TC-NER function did not reproduce the neuropathological features that we observed in *Csa^−/−^*, *Csb^−/−^*, and *Xpd^XPCS^* mice (see below). This is consistent with a broader TCR process, which encompasses transcription-coupled repair of non-NER/non-distorting transcription-blocking lesions involving CS and TFIIH proteins.

### A marginal nervous system phenotype in GG-NER– and entirely NER–deficient mice

The GG-NER-defective *Xpc^−/−^* and total NER-defective *Xpa^−/−^* mutant mice at 26 weeks of age showed very low levels of p53-immunoreactive neurons and astrocytes, which nevertheless was higher than in *Xpd^TTD^* and wild-type mice of the same age, in which we did not detect any cells with nuclear p53 immunoreactivity throughout the nervous system. These data suggest that *Xpa^−/−^* and *Xpc^−/−^* mice have a central nervous system phenotype, albeit marginal. In case of *Xpc^−/−^* mice the phenotype is compatible with that of XP-C patients that, although neurologically and cognitively asymptomatic, may develop mild neurodegenerative changes [19]. However, in *Xpa^−/−^* mice the phenotype is very different from the severe progressive neurodegenerative changes of many XP-A patients, which develop juvenile or adult progressive neuronal degeneration throughout the central and peripheral nervous system depending on the severity of NER dysfunction [17], [19], [33], [34], [36]. Neurons from *Xpa^−/−^* mice display considerably increased sensitivity to UV radiation [77] and the cross-linking agent cisplatin [78], consistent with loss of NER function and excluding redundancy of NER activity by other proteins at least for the lesions induced by these agents. The discrepancies between human and rodents may follow from differences in the rate of production and type of DNA lesions caused by endogenous metabolites, and from the shorter lifespan of mice.

### Synergistic effects of NER and TCR deficiencies in neuronal degeneration

To investigate the effect of combined NER and TCR-deficiency on neuronal survival, we generated a Cre-lox-based conditional *Xpa* mouse model to inactivate *Xpa* selectively in postnatal neurons in *Csb^−/−^* mice. The use of a conditional *Xpa* mouse model was required in view of our previous findings that global *Csb^−/−^/Xpa^−/−^* double mutant animals show degenerative changes in multiple organs as well as a very short life span [62], [63], precluding prolonged analysis of neurodegeneration and separation from direct and indirect consequences. Our data show that *Csb^−/−^* mice with neuron-specific inactivation of *Xpa* develop progressive neuronal degeneration, indicating that the XPA protein (and the NER pathway as a whole) is essential for the survival of mouse neurons in the absence of the CSB protein. The time course and distribution of neurodegenerative changes indicate that the affected neurons degenerate asynchronously over a prolonged time window. When *Xpa* is inactivated in forebrain neurons of *Csb*-deficient animals, mild behavioral abnormalities were observed at 3 months of age, while death, associated with severe atrophy of forebrain areas, occurred between 12–21 months of age. Analysis of the distribution of dying neurons, as identified by active caspase 3 or ATF3 staining, showed that the level of ongoing neuronal degeneration at a given time point was low. Similarly, in *Csb*-deficient mice with selective inactivation of *Xpa* in Purkinje cells which were analyzed at a single time point, a subset of Purkinje cells had disappeared (identified as loss of calbindin staining), a very small subset was in the process degenerating or dying (ATF3 and caspase 3 staining), while a subset showed a normal appearance consistent with asynchronous degeneration. Such an asynchronous neuronal degeneration is consistent with cell death resulting from the accumulation of stochastic DNA damage [79], [80], and strongly resembles the pattern of neuronal degeneration in Ercc1^Δ/−^ mice that are impaired in several DNA repair systems, i.e. nucleotide excision repair, interstrand crosslink repair and double strand break repair [81].

Together, the data with conditional *Xpa*/*Csb*-deficient mice indicate that adult neurons in rodents are vulnerable to endogenous DNA lesions when deficient in both NER and TCR, but are able to cope with these lesions when either the TCR or NER pathway are defective. While the NER and TCR pathway share the TC-NER activity, they have non-overlapping activities consisting of GG-NER and the still poorly defined non-NER TCR activities. In neurons, factors of the GG-NER machinery, in particular XPC, have been shown to operate in a specialized type of transcription-associated repair, termed domain-associated repair (DAR). DAR operates on both strands in active genes, including regions of a gene that RNA polymerase II does not reach and has been proposed to complement TCR [80], and it may possibly mask or compensate for the loss of TCR [80]. This is supported by the demonstration that XPC-deficient mice that are selectively deficient in GG-NER when crossed with CSB-deficient mice have a similar phenotype as *Csb^−/−^Xpa^−/−^* mice [64]. Our data indicate that *Xpc^−/−^* and *Xpa^−/−^* animals develop similar marginal central nervous system phenotypes consisting of highly sporadic p53-positive cells. Together these data suggest that in mice Xpc is equally important as Xpa for the central nervous system. Also in man, XPC-deficiency may result in subtle neurodegenerative changes [19], although XPA-deficiencies results in much more severe neurodegenerative phenotypes [14], [17], [19].

Non-NER TCR has been proposed to operate in conditions of specific transcription-blocking oxidative DNA lesions, putatively via recruitment of alternative DNA repair pathways [7], [9], [10]. This explains why cells from CS mice and CS patients, unlike XPA-deficient cells, show increased vulnerability to some types of oxidative stress, and may more readily accumulate oxidative DNA lesions [9], [20], [39], [82]. In addition, increased levels of oxidative DNA lesions have been reported in brain tissue of *Csb^−/−^* mice [10]. Thus, the inability to cope with oxidative lesions may explain the pathological phenotype of CS mice, as well as the severe degenerative phenotype in the conditional *Xpa*-deficient *Csb^−/−^* mice. However, the precise identity of DNA lesions and the question whether the CS phenotype truly results from a repair deficiency remains to be further explored.

In summary, our data indicate that the GG-NER, TC-NER, and non-NER TCR mechanisms operate together in maintaining the integrity of neurons, and that the absence of one pathway aggravates the risk for deficiencies in other pathways, explaining the severe neurodegenerative phenotype in double mutants. The extent to which a combined deficiency of NER and TCR is detrimental to non-neuronal nervous systems cells remains to be determined in future studies by selectively inactivating Xpa in these cells in TCR deficient mice. We propose that neuron-specific inactivation of *Xpa*- in *Csb*-deficient mice represents a powerful model for studying XP neurological disease and the role of NER in neurons. As neurologic symptoms seen in XP are hallmark features of age-related neurodegenerative diseases these mice may also reproduce aspects of accelerated aging.

## Materials and Methods

### Mutant mice

Experiments were performed in accordance with the “Principles of laboratory animal care” (NIH publication no. 86-23) and the guidelines approved by the Erasmus University animal care committee. Animals used were *Xpc^−/−^*, knock-out for the *Xpc* gene [47], *Xpa^−/−^*, knock-out for the *Xpa* gene [41], *Csa^−/−^*, knock-out for the Csa gene [46], *Csb^−/−^*, in which the CS1AN patient mutation is mimicked resulting in a null mouse [45], *Xpd^XPCS^*, homozygous for the G602D XPCS point mutation in the Xpd gene [49] and *Xpd^TTD^*, carrying Xpd alleles with the R722W TTD mutation [50] bred in a pure C57BL/6J background.

To obtain a conditional *Xpa* knockout mouse model, we generated a targeting construct in which exon 4 was fused in frame to the mouse *Xpa* cDNA (containing the remaining coding sequence and including a synthetic polyA sequence), followed by a PGK promoter-driven hygromycin selectable marker gene (Figure 4A). A genomic clone containing 10 kb of the 129ola mouse *Xpa* locus (pMMXP3-6#13; [41]), was used to re-clone an approximately 10 kb size BamHI fragment, containing exon 3 to 6, into the psp72 vector. Following XbaI digestion, part of exon 4 and intron 4 was replaced by a cassette containing the mouse *Xpa* cDNA including the natural 3′ UTR and polyadenylation signal followed by a PGK promoter-driven hygromycin selectable marker and a LoxP site respectively. Next, the SmaI site downstream of the LoxP site was used to introduce a cassette containing a splice acceptor sequence (SA), an ochre stopcodon multiple reading frame insertion (Murfi) linker, a ribosomal entry site (IRES), and a LacZ/GFP fusion reporter gene (as a blunted SalI fragment). The SmaI site in intron 3 was used to insert a blunted XhoI-SalI loxP fragment from pGEM30 (kindly provided by Dr. W. Gu, University of Cologne). This targeting construct, which was designated pIP-Xpa-con, contains homologous arms of 4 kb at the 5′ end and 5 kb at the 3′ end.

The 129Ola-derived ES cell line IB10 was electroporated with NotI linearized pIP-Xpa-con DNA and cultured in gelatin-coated dishes as described before [45]. Hygromycin (Roche, 843555) was added 24 hr after electroporation to a final concentration of 100 µg/ml. Cells were maintained under selection for 7–8 days, after which clones were isolated and expanded in 24-well plates. Genomic DNA from individual hygromycin-resistant clones was digested with EcoRI and analyzed by Southern blotting using a 500 bp DraI fragment (“intron 5/exon 6” probe; obtained from a 7.5 kb PCR fragment spanning exon 5 and 6). EcoRI digested DNA from targeted ES clones was subsequently screened with the hygromycin (cDNA) probe to confirm proper homologous recombination at the 5′ end of the targeting construct.

For the generation of *Xpa^c/−^* ES cells, we followed the same procedure as described above, except that *Xpa^−/−^* ES cells [54] were used. To test the functionality of the loxP sites, *Xpa^c/+^* ES cells were electroporated with a purCre plasmid (kindly provided by Dr. M. Jaegle, Erasmus MC) and cultured on gelatin dishes as described. Puromycin (Sigma, P7255) was added 24 hr after electroporation to a final concentration of 100 µg/ml. Cells were maintained under selection for 3 days. Genomic DNA from individual puromycin-resistant clones was digested with EcoRV and analyzed by Southern blotting using a 500 bp PCR fragment of the LacZ gene. Properly targeted IB10 ES clones were karyotyped and cells from two independent clones (selected for the presence of 40 chromosomes) were injected into 3.5-day-old C57BL/6J blastocysts. Male chimeric mice were mated with C57BL/6J females to obtain heterozygote offspring.

Heterozygous males and females were bred to *Xpa^+/−^* as well as *Csb^−/+^* animals to ultimately obtain *Xpa^c/−^*, *Xpa^c/+^* and *Csb^−/−^/Xpa^c/−^* animals. Genotyping was initially performed by Southern blot analysis of genomic DNA obtained from tail biopsies of 8–10-day-old born pups. A description of PCR-based genotyping methods is given below. *Xpa^c/−^* and *Csb^−/−^*/*Xpa^c/−^* animals were also interbred with *Cag-Cre* [65], *CamKII-Cre* (line L7ag#13) [66], [67], and *L7-Cre* (line L7*Cre-2*, [71]) Cre-recombinase transgenic mice, which were kindly provided by A. de Wit (ErasmusMC), S. Zeitlin (Columbia University), and J.J. Barski (Max-Planck-Institute of Neurobiology, Martinsried, Germany), respectively.

Primary mouse embryonic fibroblasts from the various single and double mutant mouse models (three independent lines per genotype) were isolated from day 13.5 embryos and cultured as described before [83].

Mice and cells were genotyped by PCR for the wild-type and (conditional) mutant *Xpa* or *Csb* alleles using a primer mix that (per genotype) amplifies both the wild-type and targeted alleles in a single reaction [41]. The presence or absence of the conditional *Xpa* allele was detected by PCR using primers *Xpa*Fex3 (5′-TTT GAT CTG CCA ACG TGT G-3′) and *Xpa*Rex4 (5′-GCT TCG CTT CTG TCT TGG T-3′). The presence or absence of the *Cre* transgene was detected by PCR using primers 5′-GCA CGT TCA CCG GCA TCA AC-3′ and 5′-CGA TGC AAC GAG TGA TGA GGT TC-3′. Both products were amplified with the same PCR program: 5 min. 93°C, 1 min. 93°C, 1 min. 58°C, 2.5 min. 72°C (35 cycles of the latter three steps), 5 min. 72°C.

### LacZ staining of embryos and cells

Cells or embryos were fixed for 30 minutes at 4°C in a buffer containing 1% paraformaldehyde, and subsequently washed 3×15 minutes with PBS/0.01% NP40. Cells or embryos were stained overnight at 37°C in dark in a staining solution containing 3.1 mM K_3_Fe(CN)_6_, 3.1 mM K_4_Fe(CN)_6_, 0.15 M NaCl, 1 mM MgCl_2_ and 1 mg/ml X-gal (Roche Applied Sciences, USA, IN). For tissues, the same procedure was used, except that the fixation time was extended to 3 hours.

### DNA repair assays

Seeded cultures at a density of 1000 spontaneously immortalized MEFs on a 6 cm dish were exposed to different doses of UV-C (254 nm, Philips TUV lamp). The cells were allowed to grow for another 7 days after which the resulting clones were fixed, stained and counted. For each independent cell line, the amount of surviving clones at each dose of UV, 3 dishes per dose, was calculated as the percentage of clones on the plate without UV.

### Behavioral assays

For the open field test, animals were placed for 30 min in a square (26×26×26 cm) open field box, equipped with photobeam sensors (TruScan E63 10–12, Coulbourn Instruments), and attached to a computer to record the following ambulatory parameters: total distance, center distance, total move time, center time and corner time. Each test session lasted 30 minutes, and data were collected in 5 minute intervals. The anxiety ratio was calculated by dividing center distance by total distance. Rotarod analyses were performed as described previously [84].

### Immunohistochemical and histopathological procedures

Mice were anesthetized with pentobarbital and perfused transcardially with 4% paraformaldehyde, and brains were dissected out, weighed, and postfixed overnight in 4% paraformaldehyde at 4°C. For standard histological analyses brains were paraffin-embedded, sectioned at 4 µm and stained with haematoxylin/eosin solution. For other staining procedures brain specimen were embedded in gelatin blocks [81] and sectioned at 40 µm with a freezing microtome. Sections were processed, free floating, using immunofluorescence or a standard avidin-biotin–immunoperoxidase complex method (ABC; Vector Laboratories) with diaminobenzidine (0.05%) as the chromogen. In addition, a selected number of frozen sections were processed for a silver staining procedure that selectively labels dying neurons and their processes [81].

Immunoperoxidase-stained sections were analyzed and photographed using a Leica (Nussloch, Germany) DM-RB microscope and a Leica DC300 digital camera. Sections stained for immunofluorescence were analyzed with a Zeiss (Oberkochen, Germany) LSM 510 confocal laser scanning microscope using 40x/1.3 and 63x/1.4 oil-immersion objectives.

Primary antibodies reported in this study are as follows: mouse anti-APC (Calbiochem, clone CC-1, 1∶2000); rabbit anti-activating transcription factor 3 (ATF3; Santa Cruz Biotechnology, Santa Cruz, 1∶1000); rabbit anti-cleaved caspase 3 (Asp175; Cell Signaling Technology, 1∶200); mouse anti-calbindin (Sigma, clone CB-955, 1∶10000); rabbit anti-GFAP (DAKO, 1∶5000); mouse anti-GFAP (Sigma, clone G-A-5, 1∶20000); rabbit anti-HSP25 (Stressgen, 1∶7000); rabbit anti-Iba1 (WAKO Chemicals, 1∶ 2000); rat anti-Mac2 (Cedarlane, 1∶2000); mouse anti-MAP2 (Millipore, clone AP20, 1∶200); rat anti-myelin basic protein (Millipore, MAB386, 1∶500); mouse anti-NeuN (Millipore MAB377, 1∶2000); rabbit anti-neurofilament-H (Millipore, 1∶2000); rabbit anti-olig2 (IBL, 1∶2000); rabbit anti-p53 (Leica, 1∶2000); mouse anti-S100B (Sigma, clone 1B2, 1∶2000); and guinea pig anti-VGLUT1 (Millipore, 1∶2000). For avidin-biotin–peroxidase immunocytochemistry biotinylated secondary antibodies from Vector Laboratories (Burlingame, CA) diluted 1∶200 were used. FITC-, cyanine 3 (Cy3)-, and Cy5-conjugated secondary antibodies raised in donkey (Jackson ImmunoResearch, West Grove, PA) diluted at 1∶200 were used for immunofluorescence.

Immunoperoxidase-stained sections were analyzed and photographed using a Leica DM-RB microscope and a Leica DC300 digital camera. To determine the relative staining intensity of GFAP staining, sections were photographed using a 5× objective, and optical densities were determined from TIFF files using MetaMorph 4.6 image analysis software. Optical densities determined in rectangular areas of 200×250 µm. To minimize variability resulting from the staining procedure this analysis was performed with sections stained in a single immunostaining session.

### Statistical analyses

Statistical analyses were done with GraphPad Prism software (San Diego, USA). Means from different age groups, and different transgenic mouse lines were compared using one-way-ANOVA with Tukey's post tests.

## Supporting Information

Figure S1 P53 expression in brain and spinal cord of adult NER– and TCR–deficient mice. Double-labeling confocal images showing p53-NeuN (A, H, I), p53-S100 (B), p53-GFAP (C, D, F, G), and p53-APC (E) double-labeling in cerebellar cortex, the mesencephalic reticular formation and spinal cord of NER-deficient mice. Note in A three p53-immunoreactive cells, two of which (the cell indicated by the arrow and the cell shown in A″) are cerebellar granule cells. Also note p53-positive astrocytes with large intensely p53-immunoreactive nucleus (A′), or with nuclei with a DAPI-negative center that are intensely immunoreactive for p53 (arrow in B′, C). The nucleus of the p53-immunoreactive astrocyte shown in G also shows abnormal DAPI staining. Panel (I) illustrates an intensely p53-immunoreactivity motor neuron in the spinal cord of an *Xpd^XPCS^* mouse. Notably, p53 immunoreactive neurons, while occasionally present in spinal cord and brain stem of *Xpd^XPCS^* mice were never observed in spinal cord and brainstem of *Csa^−/−^* and *Csb^−/−^* mice, illustrating subtle differences between these mouse lines. Scale bars: 25 µm (A, B, I), 20 µm (E) and 10 µm (C, D, F, G, H).
(TIF)

Figure S2 Microglia activation in spinal cord of Cockaybe syndrome mutant mice. Double-labeling confocal images of Iba-1 and Mac2 staining in spinal cords of 25 week old wild-type, *Csa^−/−^*, *Csb^−/−^*, *Xpa^−/−^*, *Xpc^−/−^*, *Xpd^XPCS^*, and *Xpd^TTD^* mice showing Mac2-positive microglia in spinal cords of *Csa^−/−^*, *Csb^−/−^* and *Xpd^XPCS^* mice (arrows in B, C, D, G). All Mac2-positive microglia cells show an activated morphology as revealed by Iba-1 staining (D). DH, dorsal horn; IZ, intermediate zone; lf, lateral funiculus; vf, ventral funiculus. Scale bar in A, 250 µm.
(TIF)

Figure S3 TCR–deficient mice show microglia activation in the white matter. Photomicrographs of Mac2-stained transverse brain sections of a 25 week old wild-type and *Csb^−/−^* mouse showing a high concentration of Mac2-positive microglia cells occurs throughout the nervous system of *Csb^−/−^* mouse (E–H). Individual or clustered Mac2-positive microglia cells are concentrated in regions containing myelinated axons such as the corpus callosum (cc), the internal capsule (ic) bundles in the striatum, the fimbria fornix (f), the cerebellar white matter, the mesencephalic (MesRF), and medullary reticular formation (RF). No or a low number of Mac2-immunoreactive cells occur in grey matter regions, such as the Neocortex NCx), the hippocampal subfields (DG [dentate gyrus], CA1 and CA3), and the medial (MVe) and lateral (LVe) vestibular nuclei. Scale bar in A: 200 µm.
(TIF)

Figure S4 Increased GFAP-and Hsp25 immunoreactivity in Cockayne syndrome mutant mice. A–Q) Photomicrographs of GFAP-immunoperoxidase staining in transverse sections at the level of caudal hippocampus and mesencephalon (A–E), the cerebellum and the medulla oblongata (F–J), and spinal cord (O–Q). Note increased GFAP-staining in mesencephalic (MesRF, arrows in B–D), and medullary (G′–I′) reticular formation of *Csa^−/−^*, *Csb^−/−^* and *Xpd^XPCS^* mice as compared to other genotypes. Also note increased GFAP-immunoreactivity in spinal cord (P, Q), while no changes in GFAP staining occurred in cerebellar cortex (L) and neocortex (N) of Cockayne syndrome mice. In white matter areas such as the corpus callosum there was no distinguishable increase in GFAP-immunoreactivity in Cockayne syndrome mice as compared to wild-type. This may be explained by relatively high levels of baseline GFAP-immunoreactivity in white matter in wild-type. R–V) Photomicrograph and bar graph illustrating the presence of Hsp25-immunoreactive astrocytes in reticular formation and cerebellar white matter of *Csa^−/−^*, *Csb^−/−^* and *Xpd^XPCS^* mice. Hsp25 immunoreactivity is also associated with endothelial cells in both wild-type and *Csb^−/−^* sections. Values in bar graph are means ± SE of 3 mice based on analysis of three sections per mouse. Scale bars: 1 mm (F) and 500 µm (P), 25 µm (R).
(TIF)

Figure S5 Characterization of the conditional *Xpa* construct in Xpa-deficient ES cells. A) To test the functionality of the genomic/cDNA fusion conditional Xpa construct (Figure 4A), it was transfected in *Xpa^−/−^* ES cells [20]. Targeted *Xpa^c/−^* ES clones were detected by Southern blot analysis. DNA was digested with EcoRI and hybridized with an intron 5/exon 6 probe, external to the targeting construct. Knockout and conditional alleles gave fragments of 16 and 12 kb, respectively. B) Survival curves of *wt* (circles), *Xpa* ^−/−^ (diamonds) and *Xpa* ^c/−^ (squares) ES cells, exposed to increasing doses of UV-C light, showing that .*Xpa^−/−^* ES cells targeted with *Xpa^c^* construct displayed the same sensitivity to UV as wild-type ES cells. This indicates that the conditional *Xpa* allele fully averted the UV-hypersensitivity of *Xpa^−/−^* ES cells. Subsequent transient expression of CRE recombinase in *Xpa^c/−^* ES cells reinstated UV-hypersensitivity (data not shown).
(TIF)

Figure S6 Weight loss, reduced rotarod performance, and brain atrophy after forebrain neuron-specific knockout of *Xpa* in *Csb^−/−^* mice. A) Photograph of 9 month old male *Xpa* ^c/−^ */CamKIIα-Cre* and *Csb^−/−^/Xpa* ^c/−^/*CamKIIα-Cre* littermates, illustrating smaller appearance of the latter. B) Bar graphs of performance in an accelerating rotarod task of *CamKIIα-Cre*, *Csb^−/−^CamKIIα-Cre*, *Xpa* ^c/−^ *CamKIIα-Cre* and *Csb^−/−^*/*Xpa* ^c/−^/*CamKIIα-Cre* (n = 18) mice. * represent P<0.05, as compared to other groups of the same age and the same mice at younger age (one-way Anova with Tukey's post test). C) Brains of *Xpa* ^c/−^/*CamKIIα-Cre* and *Csb^−/−^*/*Xpa* ^c/−^/*CamKIIα-Cre* mice sacrificed at 15, 25 and 65 weeks of age; note severe atrophy of the cortex of the 65 week old *Csb^−/−^*/*Xpa* ^c/−^/*CamKIIα-Cre* mouse (white arrows).
(TIF)

Figure S7 Loss of MAP2-immunoreactivity in hippocampus after forebrain neuron-specific knockout of *Xpa* in *Csb^−/−^* mice. Double labeling confocal immunofluorescence of MAP2 and VGlut1 in the hippocampus of 16 month-old *Xpa* ^c/−^ *CamKIIα-Cre* (A) and *Csb^−/−^Xpa* ^c/−^ *CamKIIα-Cre* (B) mice. In addition to severe atrophy the hippocampus of *Csb^−/−^Xpa* ^c/−^ *CamKIIα-Cre* mice show a marked reduction of MAP2 immunoreactivity in the CA1 subfield and the dentate gyrus (DG; arrows in B1, and compare B1′ with A1′). Immunoreactivity of the vesicular glutamate transporter 1 (VGluT1), which marks a large portion of presynaptic nerve endings was unaltered, indicating that reduced labeling was specific for MAP2 (compare A2′ with B2′). Bar, 200 µm.
(TIF)
