# Supplementary figures and images for: Age-Related Neuronal Degeneration: Complementary Roles of Nucleotide Excision Repair and Transcription-Coupled Repair in Preventing Neuropathology
Source: PLoS Genet. 2011 Dec 8;7(12):e1002405. doi: 10.1371/journal.pgen.1002405 (PMC3234220; doi:10.1371/journal.pgen.1002405)

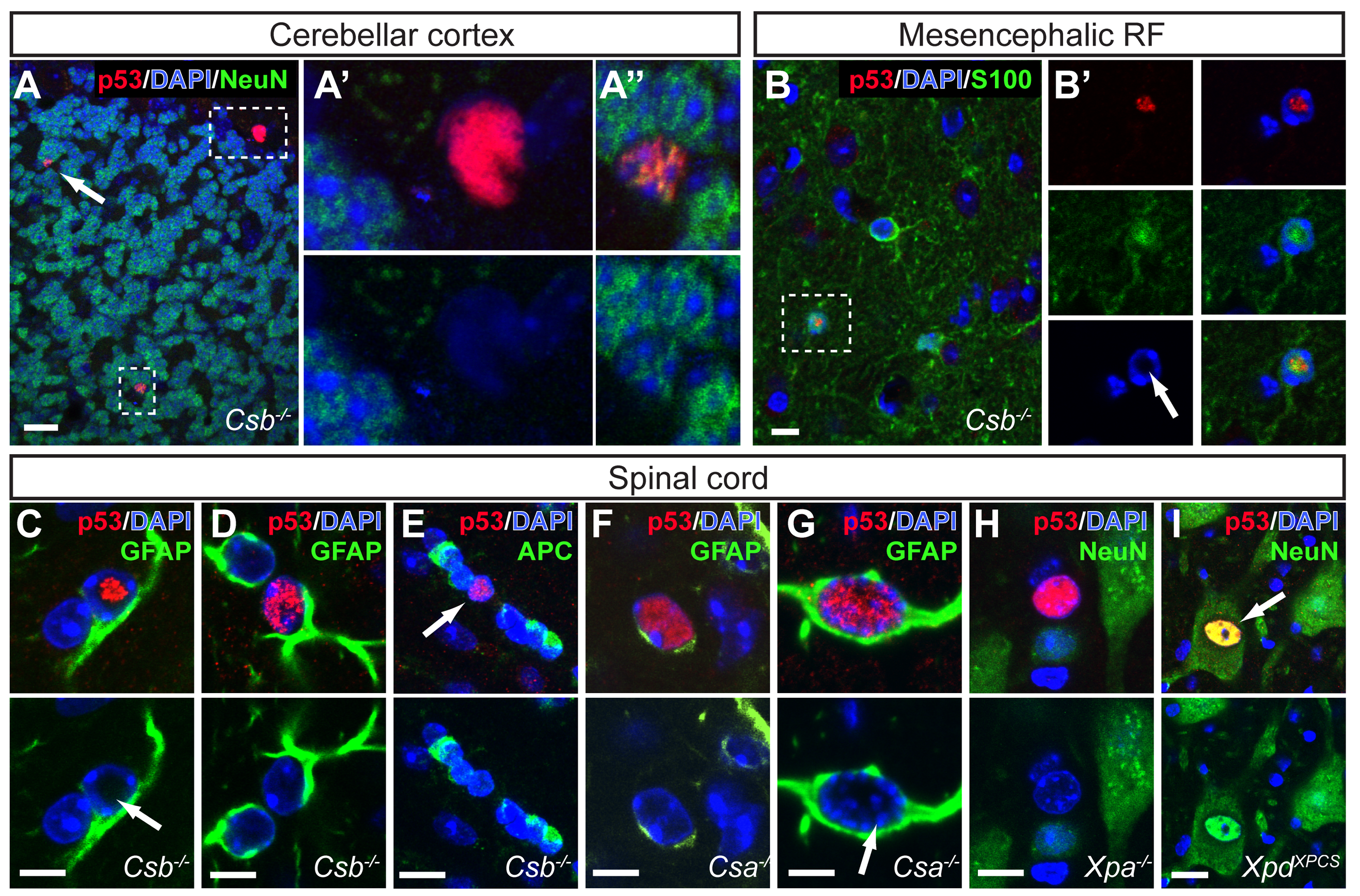

Supplement: Figure S1 — P53 expression in brain and spinal cord of adult NER– and TCR–deficient mice. Double-labeling confocal images showing p53-NeuN (A, H, I), p53-S100 (B), p53-GFAP (C, D, F, G), and p53-APC (E) double-labeling in cerebellar cortex, the mesencephalic reticular formation and spinal cord of NER-deficient mice. Note in A three p53-immunoreactive cells, two of which (the cell indicated by the arrow and the cell shown in A″) are cerebellar granule cells. Also note p53-positive astrocytes with large intensely p53-immunoreactive nucleus (A′), or with nuclei with a DAPI-negative center that are intensely immunoreactive for p53 (arrow in B′, C). The nucleus of the p53-immunoreactive astrocyte shown in G also shows abnormal DAPI staining. Panel (I) illustrates an intensely p53-immunoreactivity motor neuron in the spinal cord of an XpdXPCS mouse. Notably, p53 immunoreactive neurons, while occasionally present in spinal cord and brain stem of XpdXPCS mice were never observed in spinal cord and brainstem of Csa−/− and Csb−/− mice, illustrating subtle differences between these mouse lines. Scale bars: 25 µm (A, B, I), 20 µm (E) and 10 µm (C, D, F, G, H). (TIF) [file pgen.1002405.s001.tif]

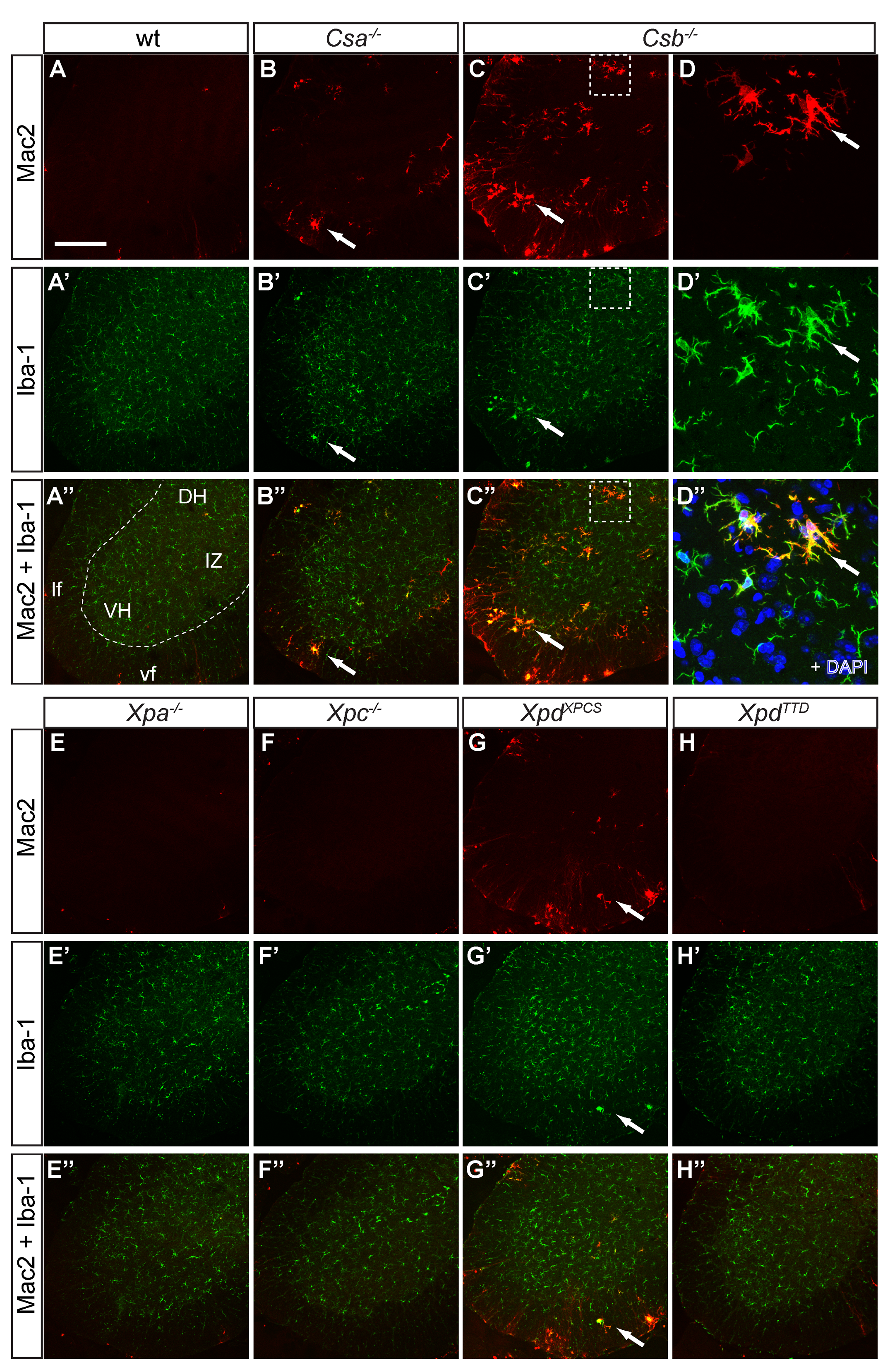

Supplement: Figure S2 — Microglia activation in spinal cord of Cockaybe syndrome mutant mice. Double-labeling confocal images of Iba-1 and Mac2 staining in spinal cords of 25 week old wild-type, Csa−/−, Csb−/−, Xpa−/−, Xpc−/−, XpdXPCS, and XpdTTD mice showing Mac2-positive microglia in spinal cords of Csa−/−, Csb−/− and XpdXPCS mice (arrows in B, C, D, G). All Mac2-positive microglia cells show an activated morphology as revealed by Iba-1 staining (D). DH, dorsal horn; IZ, intermediate zone; lf, lateral funiculus; vf, ventral funiculus. Scale bar in A, 250 µm. (TIF) [file pgen.1002405.s002.tif]

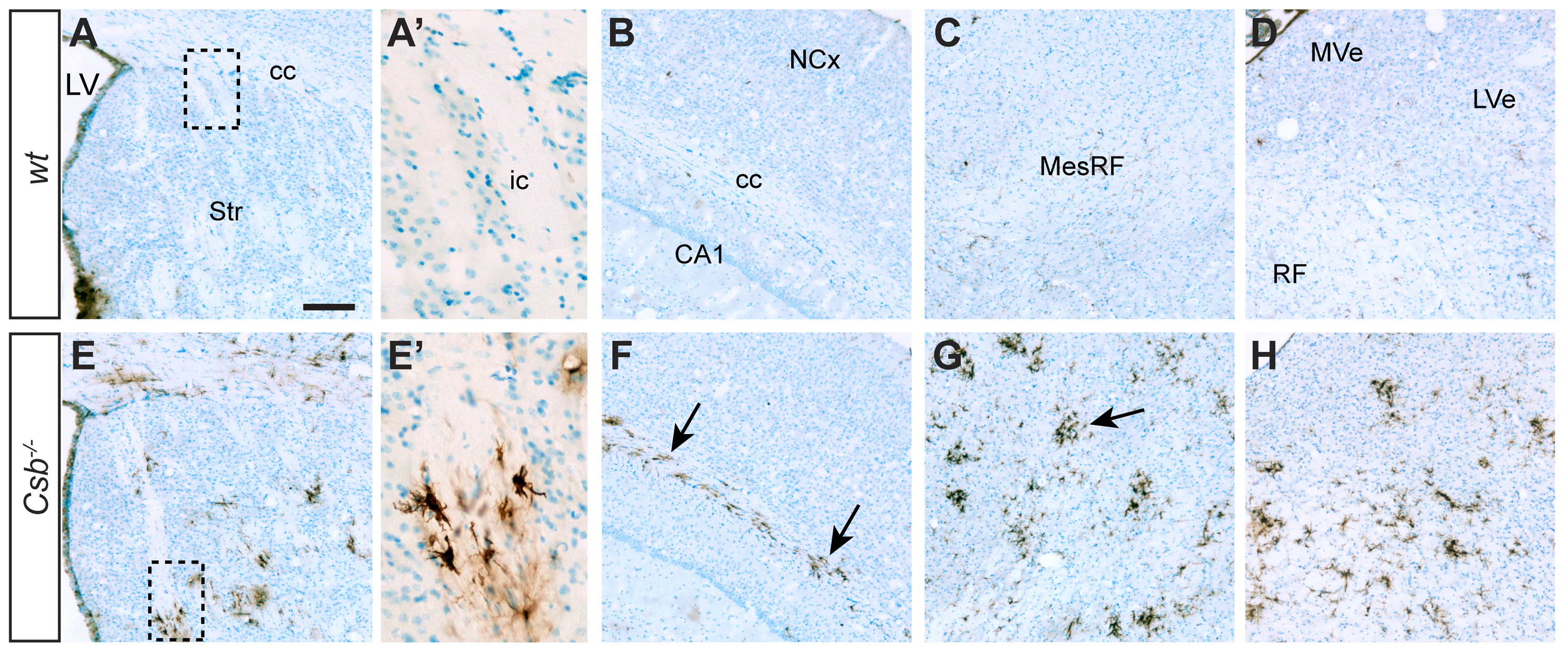

Supplement: Figure S3 — TCR–deficient mice show microglia activation in the white matter. Photomicrographs of Mac2-stained transverse brain sections of a 25 week old wild-type and Csb−/− mouse showing a high concentration of Mac2-positive microglia cells occurs throughout the nervous system of Csb−/− mouse (E–H). Individual or clustered Mac2-positive microglia cells are concentrated in regions containing myelinated axons such as the corpus callosum (cc), the internal capsule (ic) bundles in the striatum, the fimbria fornix (f), the cerebellar white matter, the mesencephalic (MesRF), and medullary reticular formation (RF). No or a low number of Mac2-immunoreactive cells occur in grey matter regions, such as the Neocortex NCx), the hippocampal subfields (DG [dentate gyrus], CA1 and CA3), and the medial (MVe) and lateral (LVe) vestibular nuclei. Scale bar in A: 200 µm. (TIF) [file pgen.1002405.s003.tif]

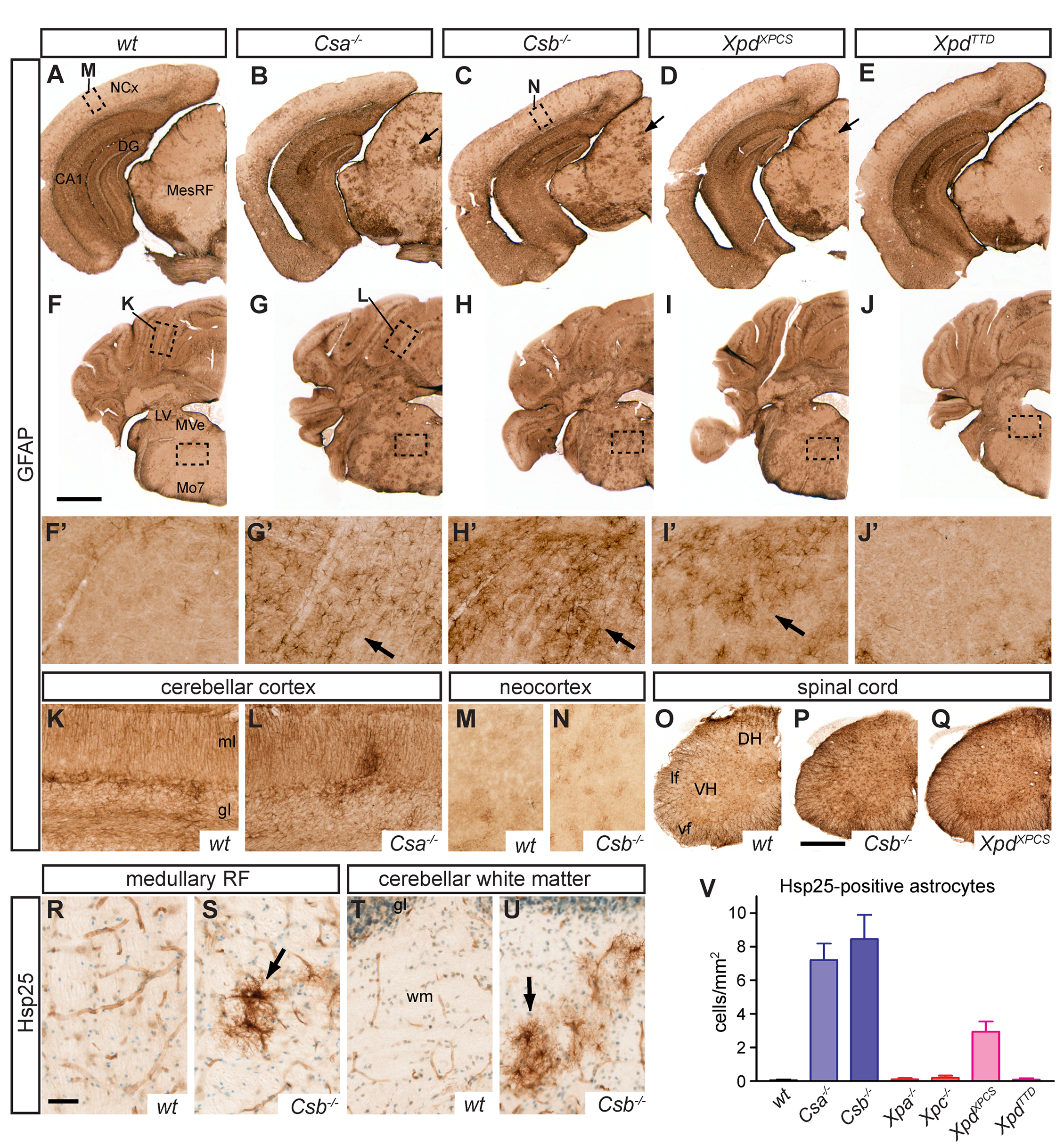

Supplement: Figure S4 — Increased GFAP-and Hsp25 immunoreactivity in Cockayne syndrome mutant mice. A–Q) Photomicrographs of GFAP-immunoperoxidase staining in transverse sections at the level of caudal hippocampus and mesencephalon (A–E), the cerebellum and the medulla oblongata (F–J), and spinal cord (O–Q). Note increased GFAP-staining in mesencephalic (MesRF, arrows in B–D), and medullary (G′–I′) reticular formation of Csa−/−, Csb−/− and XpdXPCS mice as compared to other genotypes. Also note increased GFAP-immunoreactivity in spinal cord (P, Q), while no changes in GFAP staining occurred in cerebellar cortex (L) and neocortex (N) of Cockayne syndrome mice. In white matter areas such as the corpus callosum there was no distinguishable increase in GFAP-immunoreactivity in Cockayne syndrome mice as compared to wild-type. This may be explained by relatively high levels of baseline GFAP-immunoreactivity in white matter in wild-type. R–V) Photomicrograph and bar graph illustrating the presence of Hsp25-immunoreactive astrocytes in reticular formation and cerebellar white matter of Csa−/−, Csb−/− and XpdXPCS mice. Hsp25 immunoreactivity is also associated with endothelial cells in both wild-type and Csb−/− sections. Values in bar graph are means ± SE of 3 mice based on analysis of three sections per mouse. Scale bars: 1 mm (F) and 500 µm (P), 25 µm (R). (TIF) [file pgen.1002405.s004.tif]

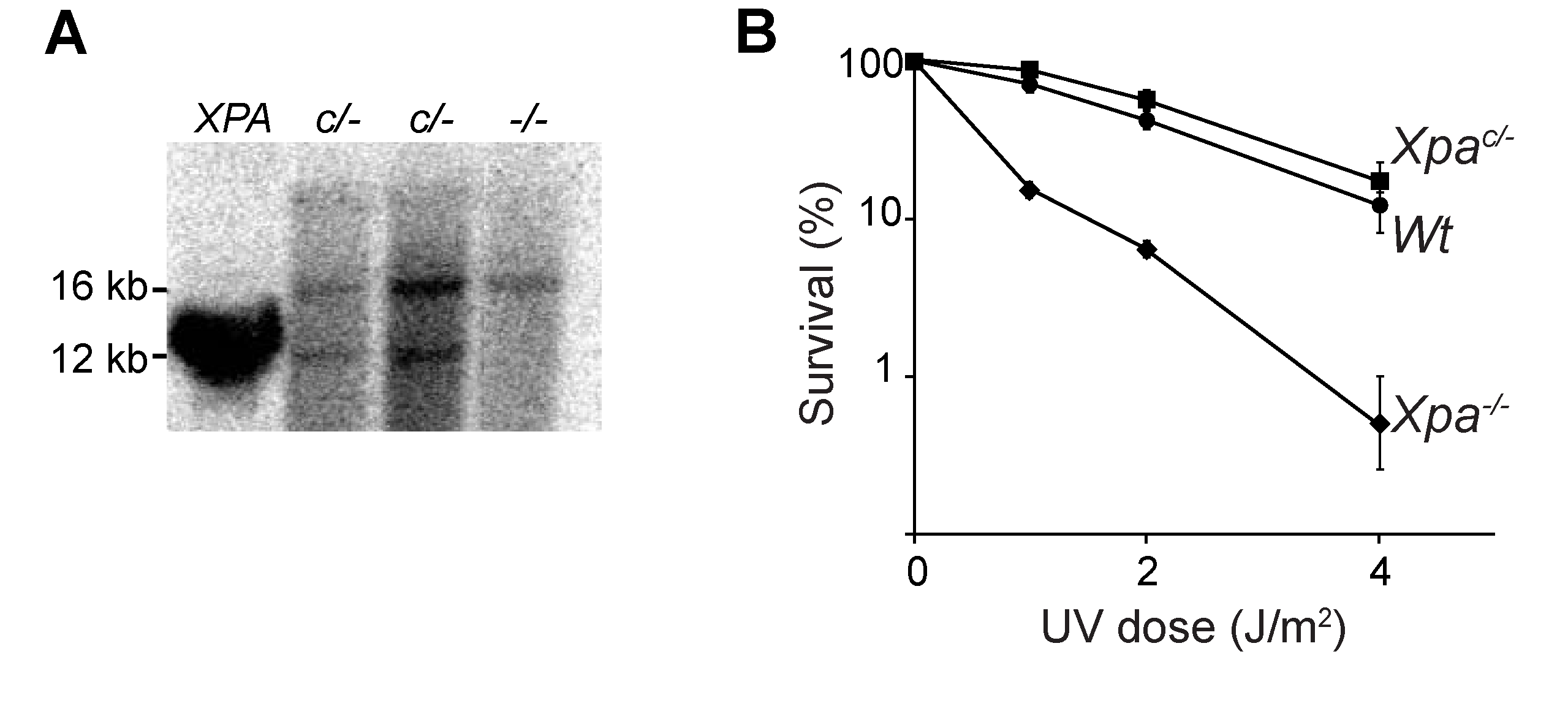

Supplement: Figure S5 — Characterization of the conditional Xpa construct in Xpa-deficient ES cells. A) To test the functionality of the genomic/cDNA fusion conditional Xpa construct (Figure 4A), it was transfected in Xpa−/− ES cells [20]. Targeted Xpac/− ES clones were detected by Southern blot analysis. DNA was digested with EcoRI and hybridized with an intron 5/exon 6 probe, external to the targeting construct. Knockout and conditional alleles gave fragments of 16 and 12 kb, respectively. B) Survival curves of wt (circles), Xpa −/− (diamonds) and Xpa c/− (squares) ES cells, exposed to increasing doses of UV-C light, showing that .Xpa−/− ES cells targeted with Xpac construct displayed the same sensitivity to UV as wild-type ES cells. This indicates that the conditional Xpa allele fully averted the UV-hypersensitivity of Xpa−/− ES cells. Subsequent transient expression of CRE recombinase in Xpac/− ES cells reinstated UV-hypersensitivity (data not shown). (TIF) [file pgen.1002405.s005.tif]

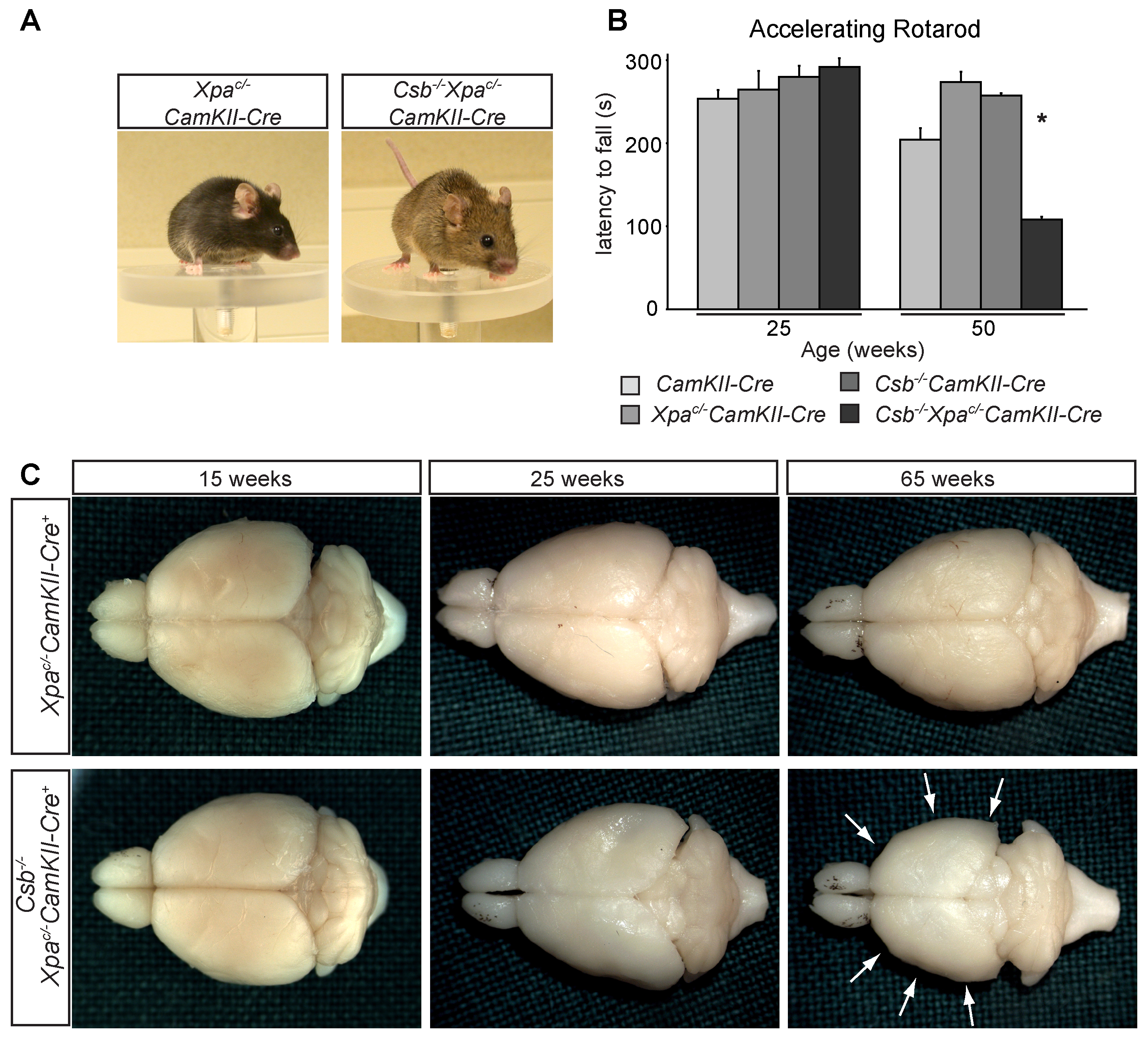

Supplement: Figure S6 — Weight loss, reduced rotarod performance, and brain atrophy after forebrain neuron-specific knockout of Xpa in Csb−/− mice. A) Photograph of 9 month old male Xpa c/− /CamKIIα-Cre and Csb−/−/Xpa c/−/CamKIIα-Cre littermates, illustrating smaller appearance of the latter. B) Bar graphs of performance in an accelerating rotarod task of CamKIIα-Cre, Csb−/−CamKIIα-Cre, Xpa c/− CamKIIα-Cre and Csb−/−/Xpa c/−/CamKIIα-Cre (n = 18) mice. * represent P<0.05, as compared to other groups of the same age and the same mice at younger age (one-way Anova with Tukey's post test). C) Brains of Xpa c/−/CamKIIα-Cre and Csb−/−/Xpa c/−/CamKIIα-Cre mice sacrificed at 15, 25 and 65 weeks of age; note severe atrophy of the cortex of the 65 week old Csb−/−/Xpa c/−/CamKIIα-Cre mouse (white arrows). (TIF) [file pgen.1002405.s006.tif]

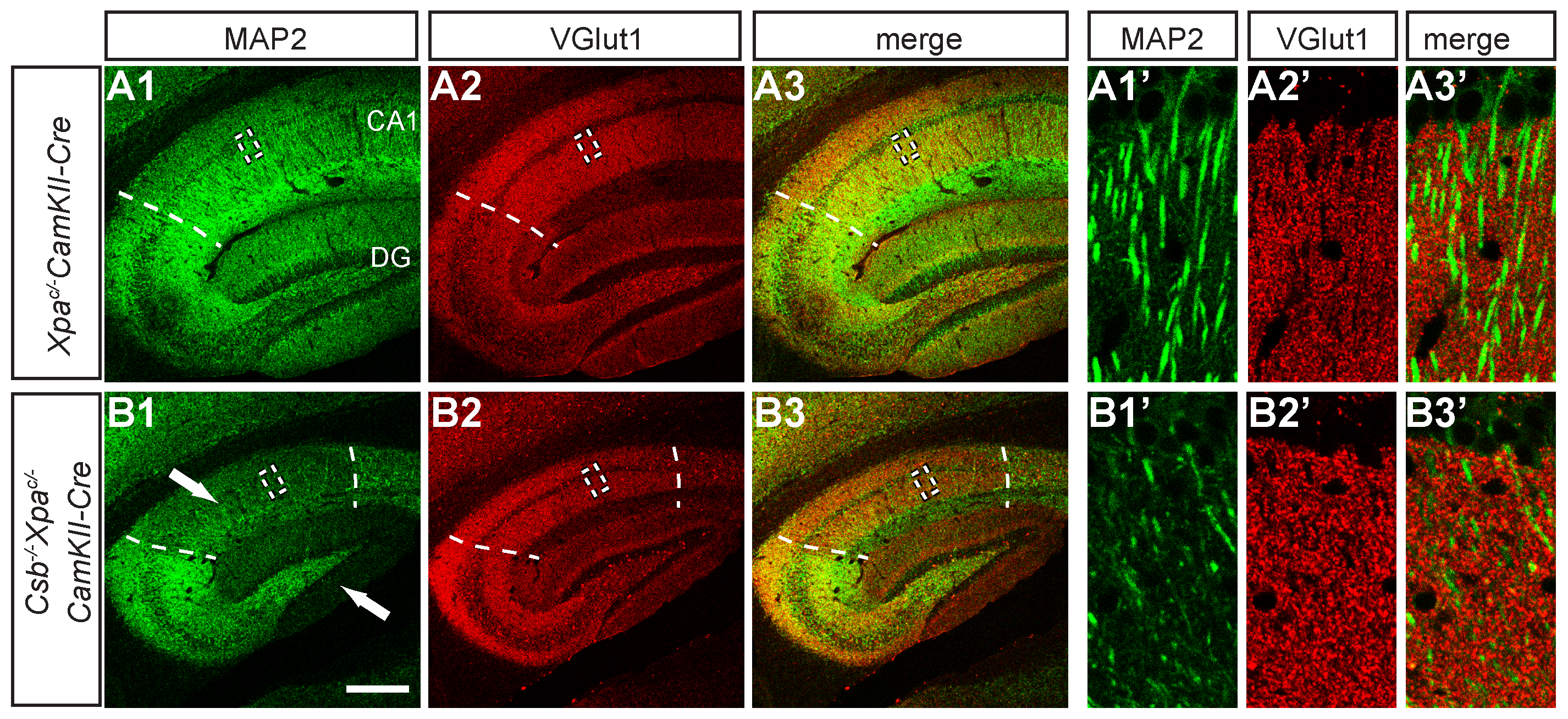

Supplement: Figure S7 — Loss of MAP2-immunoreactivity in hippocampus after forebrain neuron-specific knockout of Xpa in Csb−/− mice. Double labeling confocal immunofluorescence of MAP2 and VGlut1 in the hippocampus of 16 month-old Xpa c/− CamKIIα-Cre (A) and Csb−/−Xpa c/− CamKIIα-Cre (B) mice. In addition to severe atrophy the hippocampus of Csb−/−Xpa c/− CamKIIα-Cre mice show a marked reduction of MAP2 immunoreactivity in the CA1 subfield and the dentate gyrus (DG; arrows in B1, and compare B1′ with A1′). Immunoreactivity of the vesicular glutamate transporter 1 (VGluT1), which marks a large portion of presynaptic nerve endings was unaltered, indicating that reduced labeling was specific for MAP2 (compare A2′ with B2′). Bar, 200 µm. (TIF) [file pgen.1002405.s007.tif]
